# Supplementary material for: Design, synthesis, and biological evaluation of phenylisoxazole-based histone deacetylase inhibitors
Source: PLoS One. 2025 Nov 5;20(11):e0334632. doi: 10.1371/journal.pone.0334632 (PMC12588503; doi:10.1371/journal.pone.0334632)
Supplement: S1 File — (DOCX) [file pone.0334632.s001.docx]

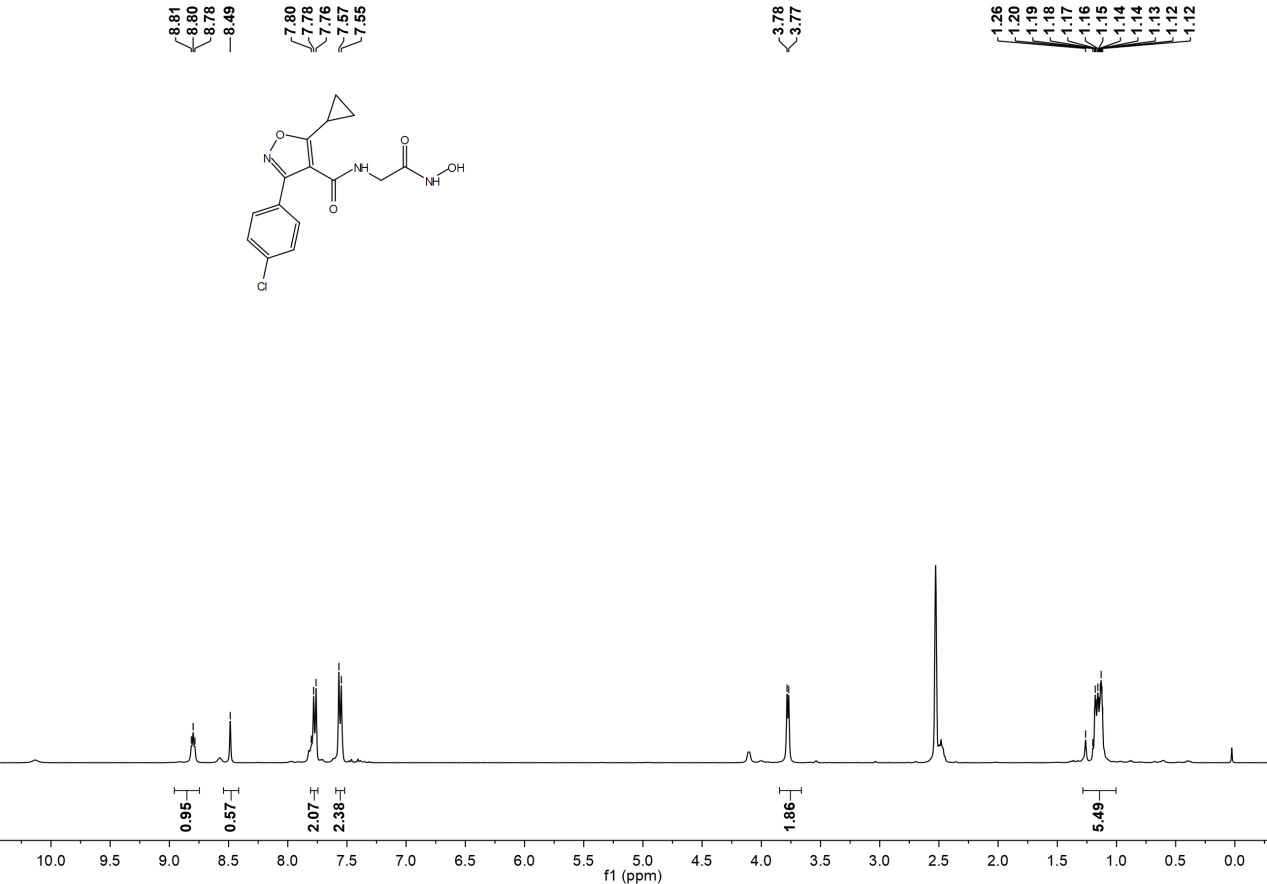


^1^H-NMR of compound **7**
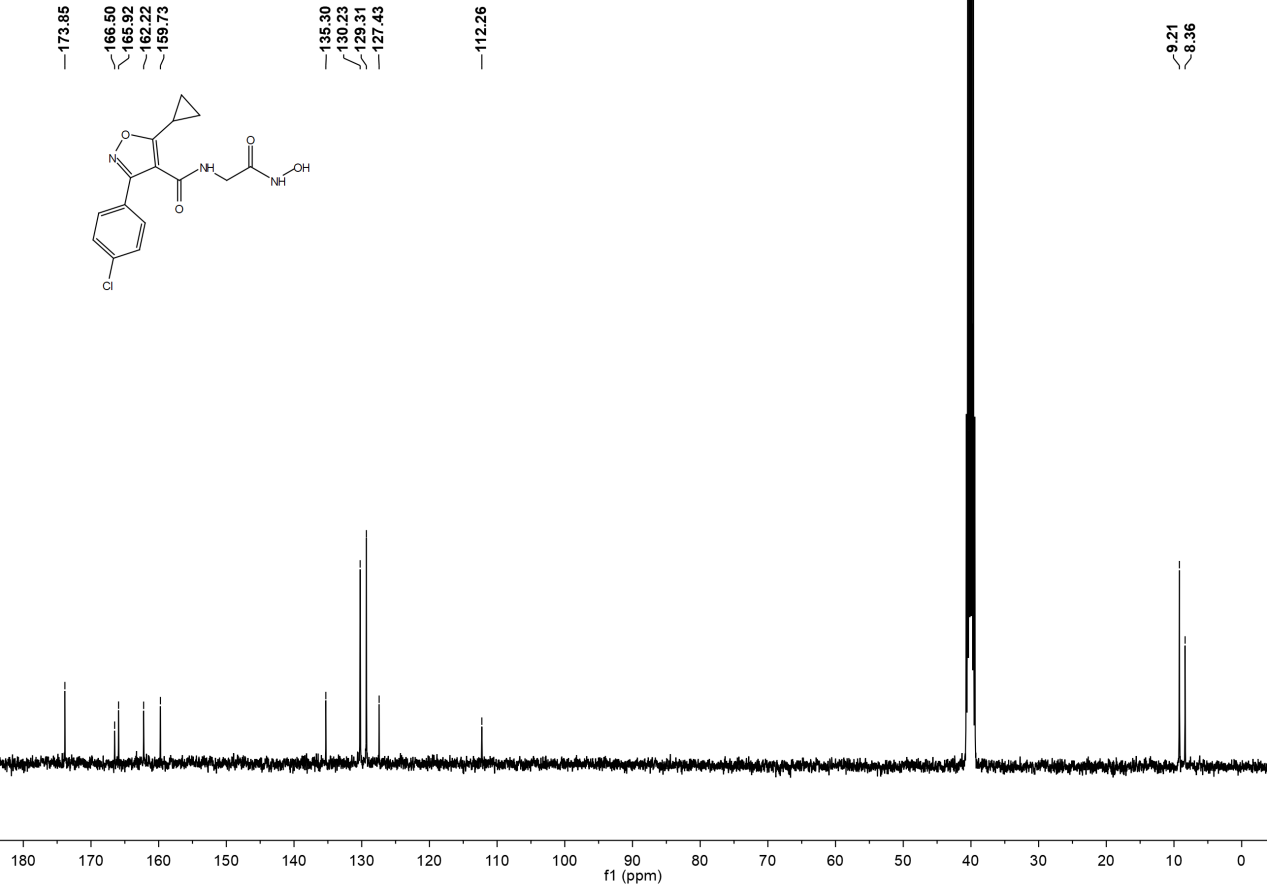


^13^C-NMR of compound **7**

Mass spectra of compound **7**


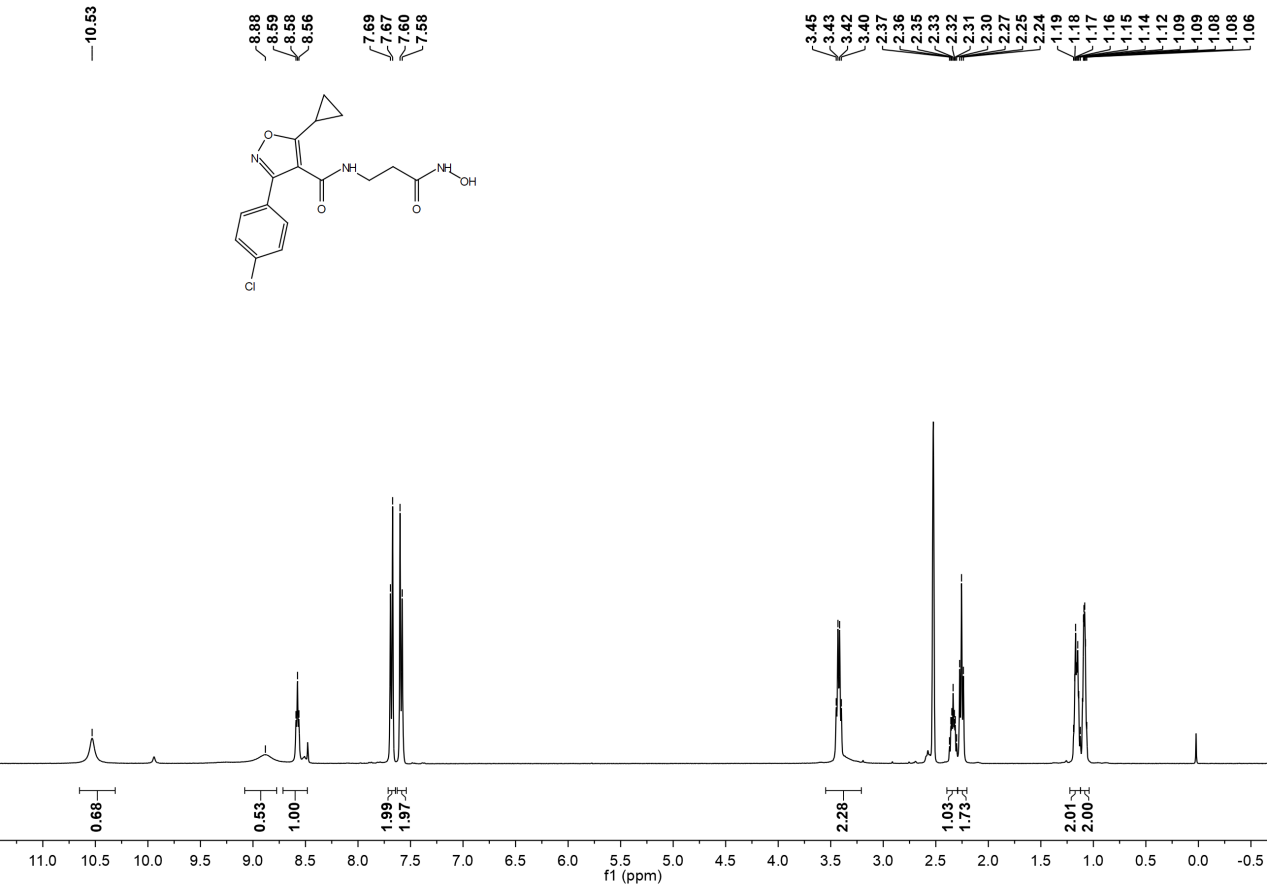


^1^H-NMR of compound **8**


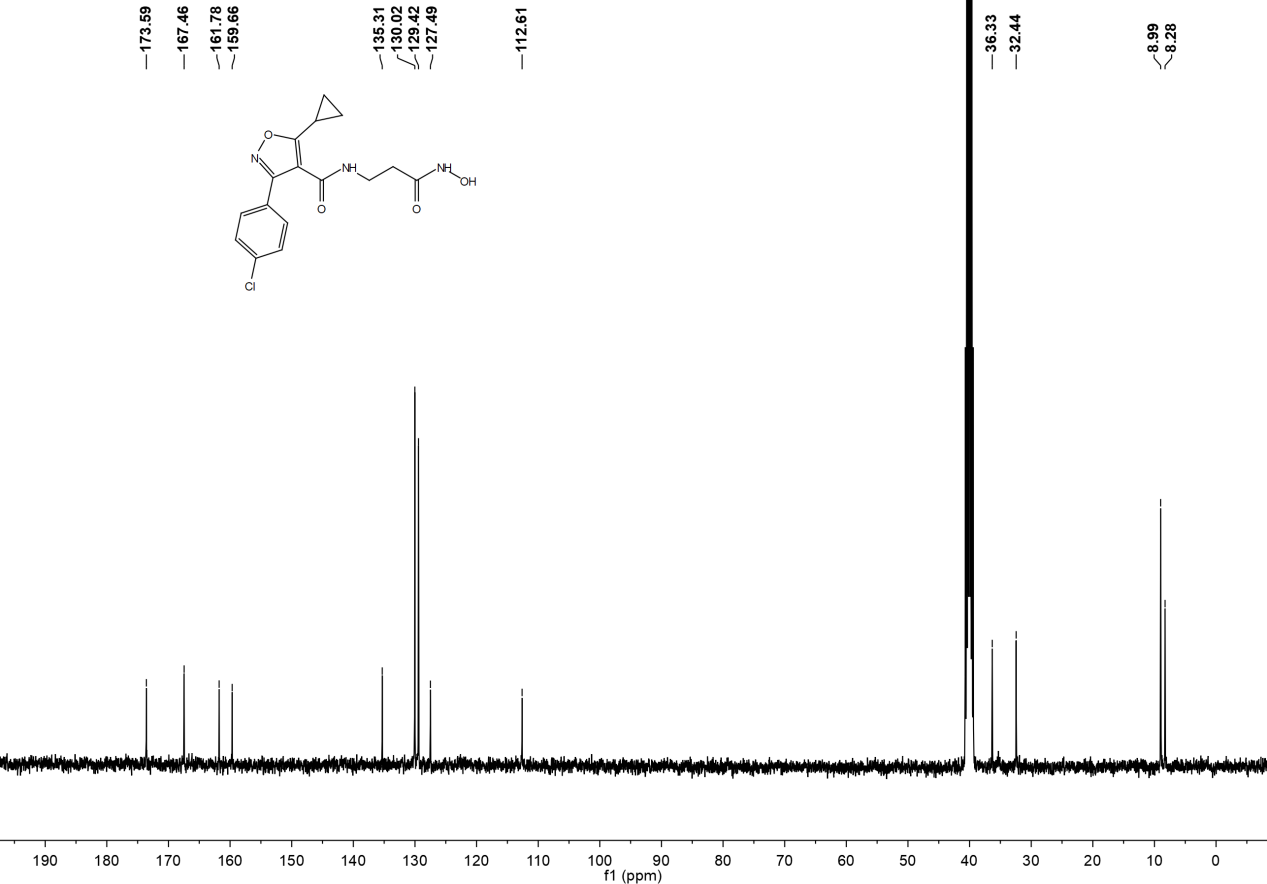


^13^C-NMR of compound **8**

Mass spectra of compound **8**
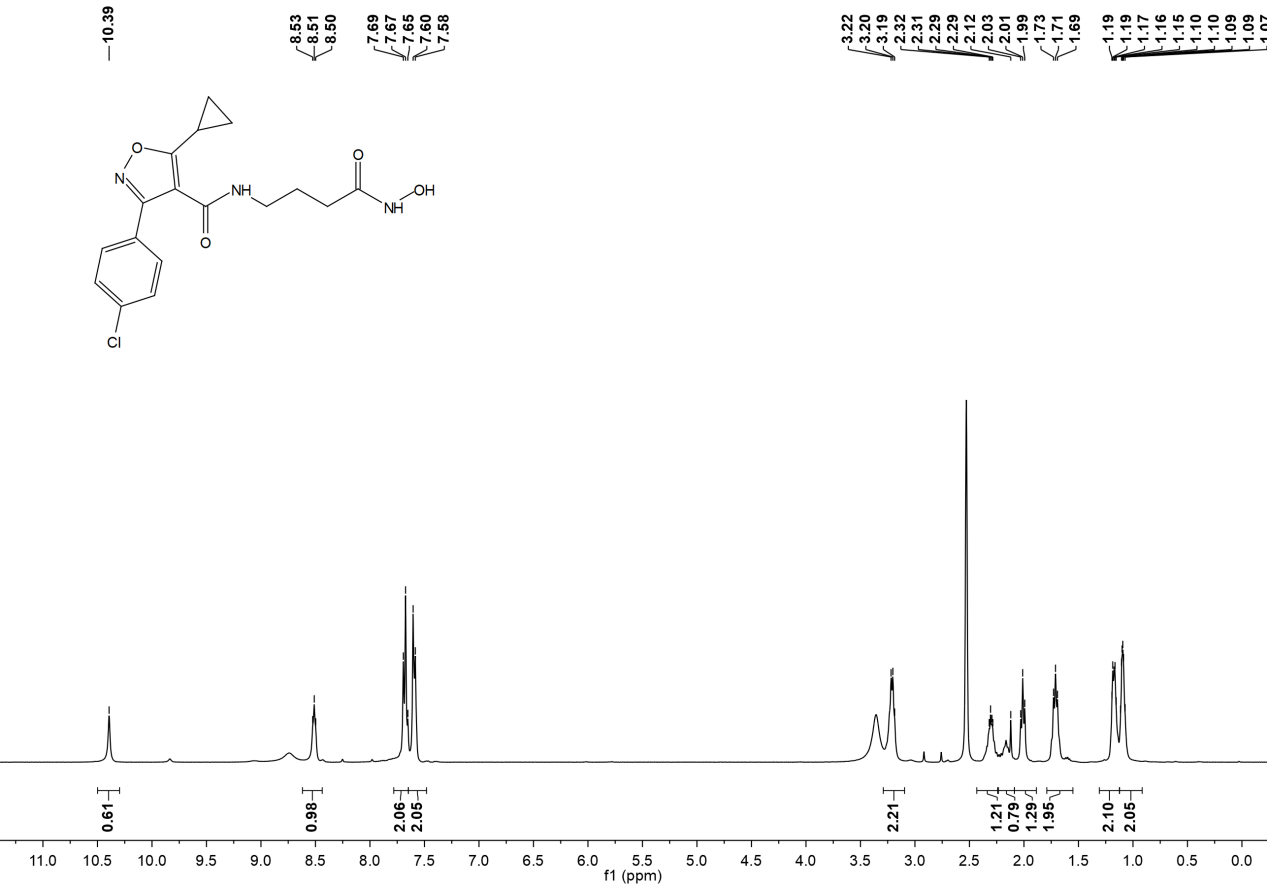


^1^H-NMR of compound **9**


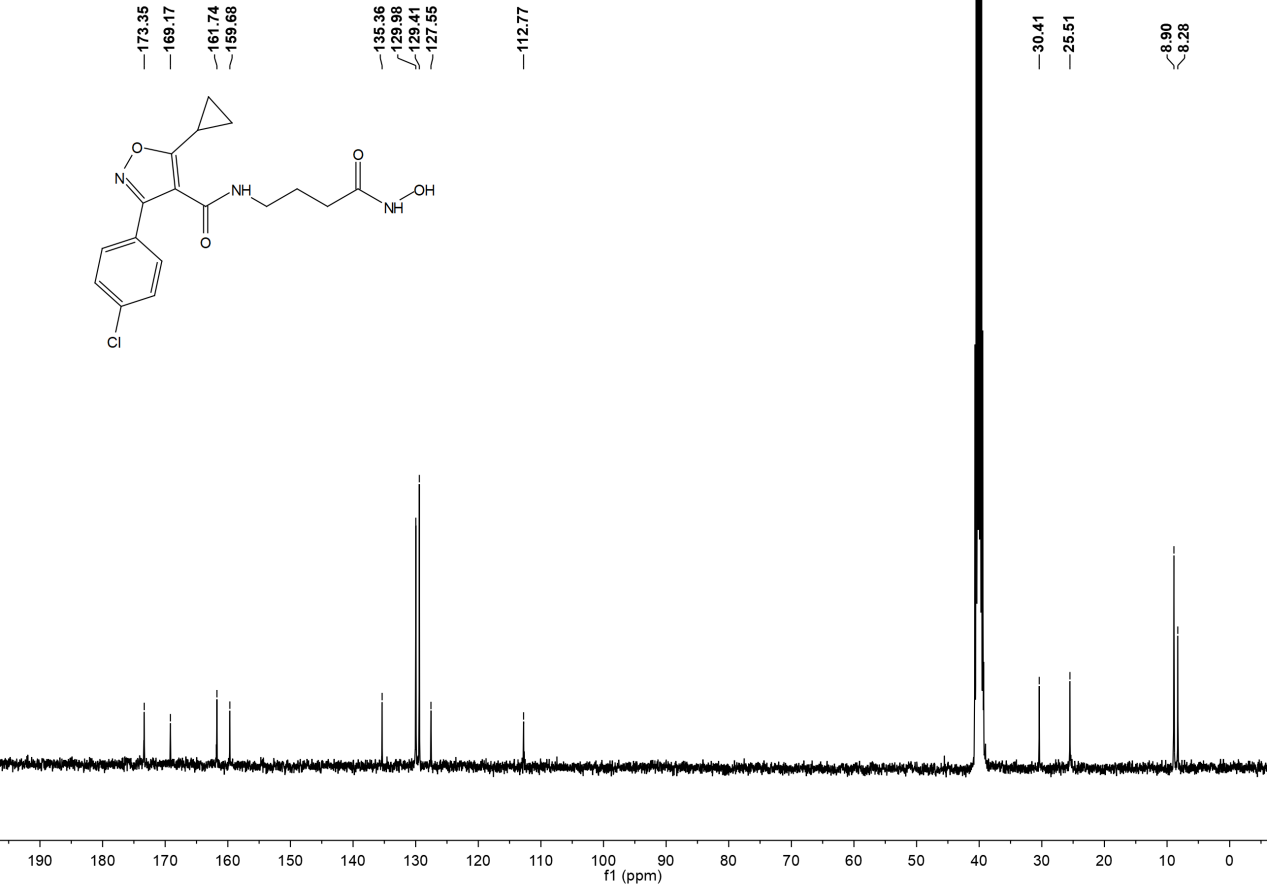


^13^C-NMR of compound **9**

Mass spectra of compound **9**


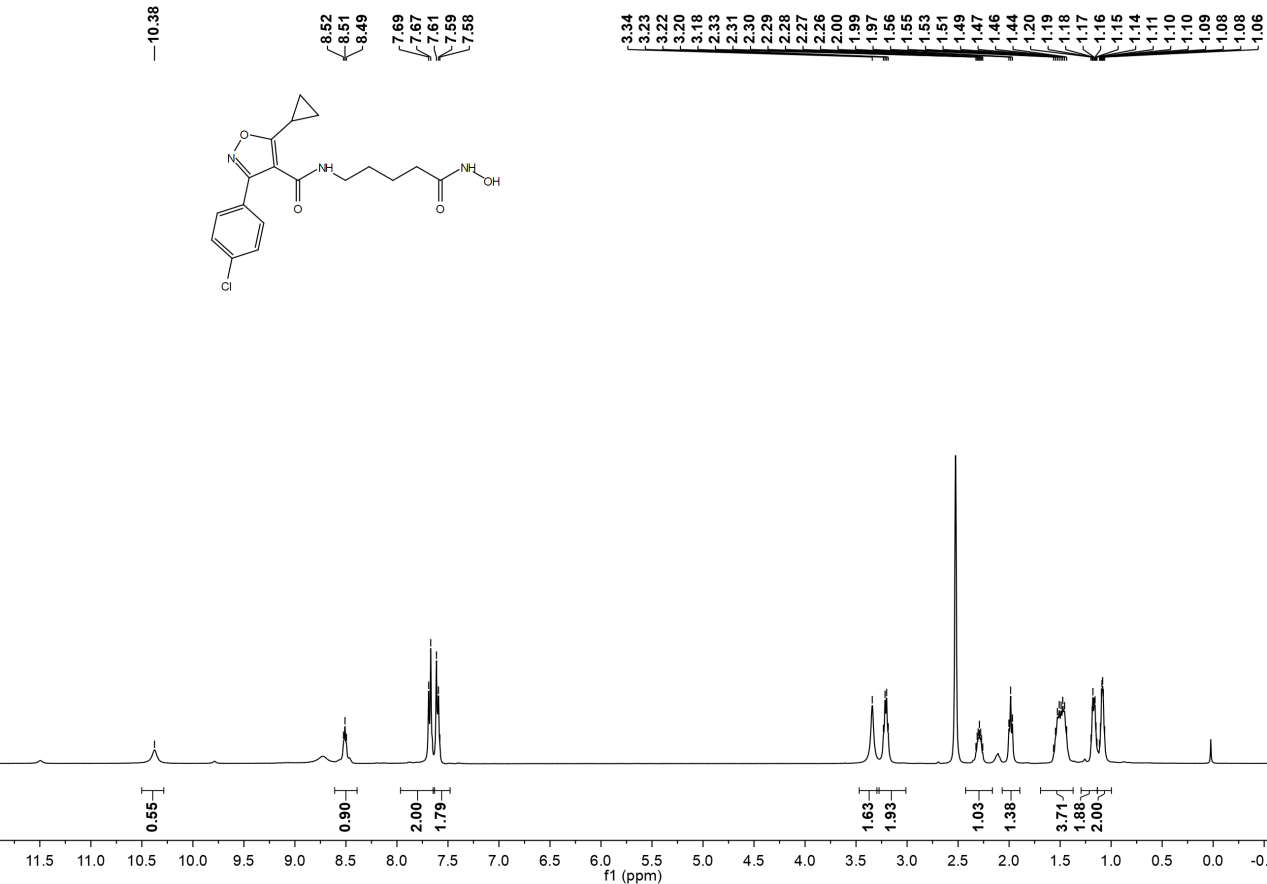


化^1^H-NMR of compound **10**


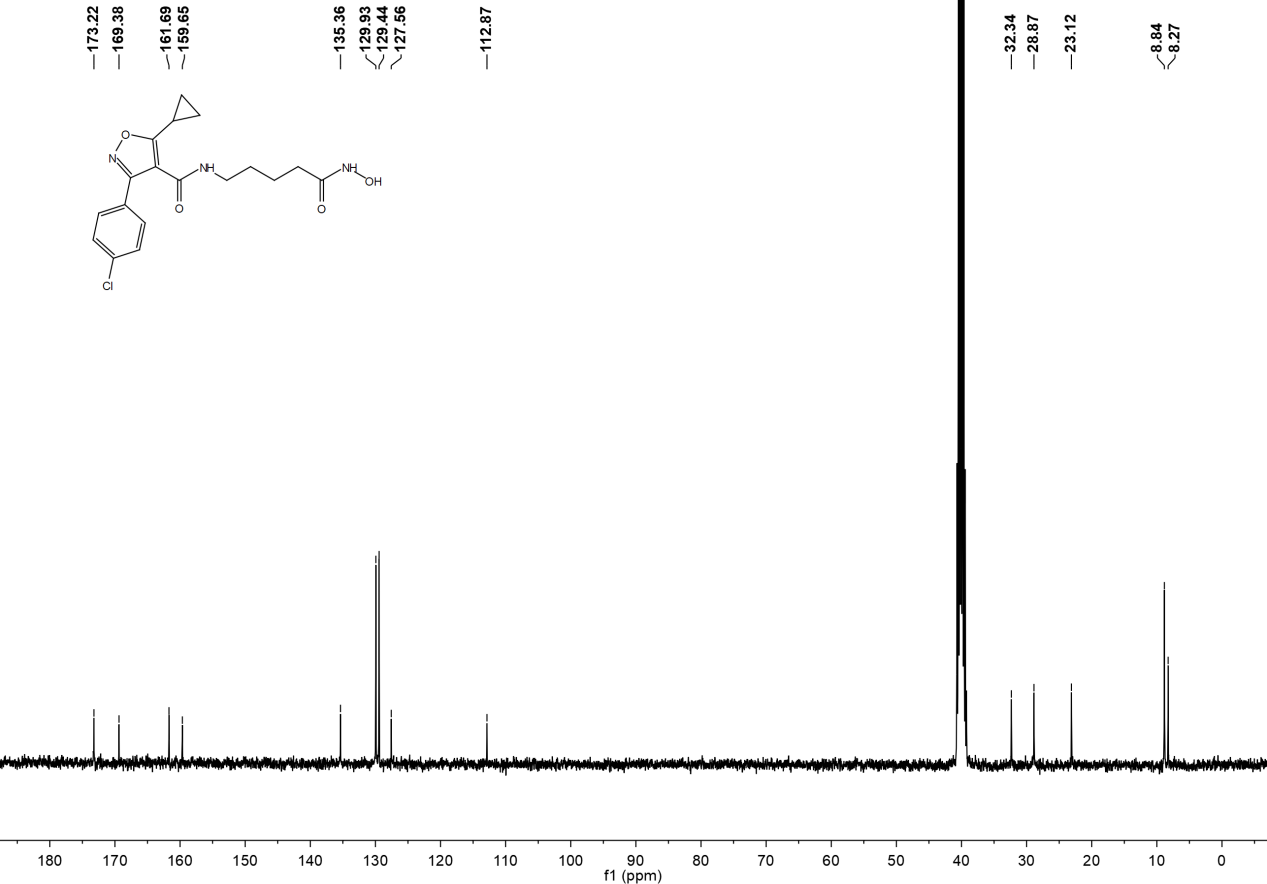


^13^C-NMR of compound **10**

Mass spectra of compound **10**


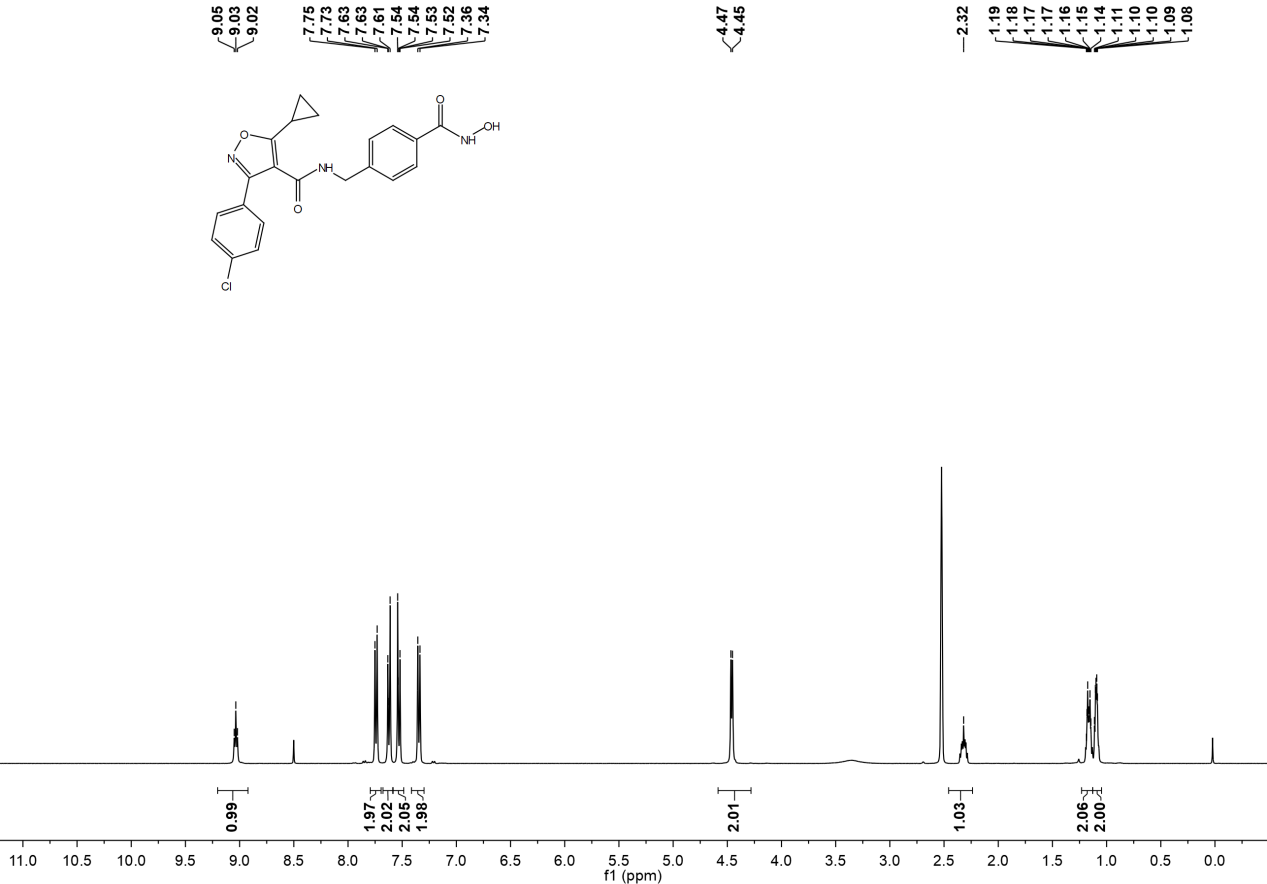


^1^H-NMR of compound **11**


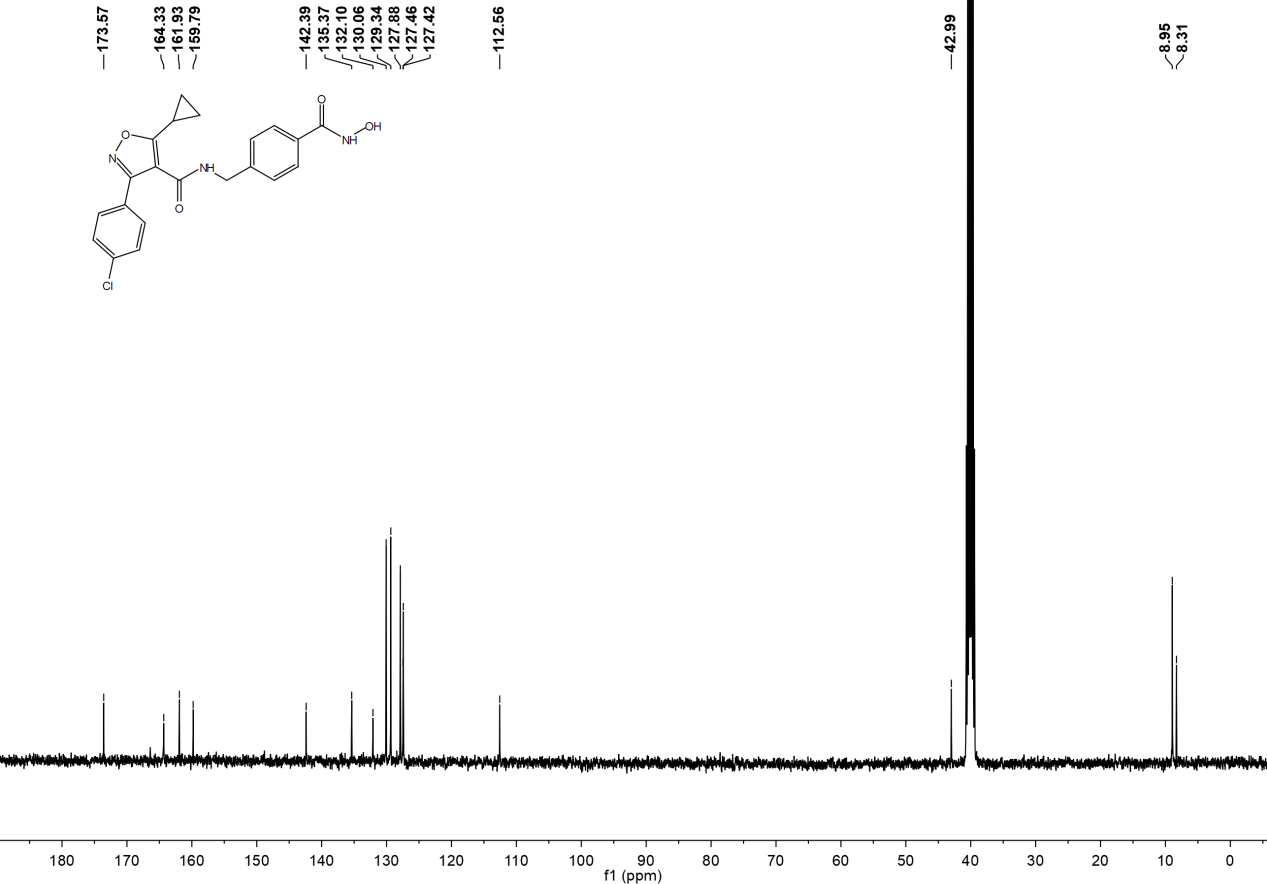


^13^C-NMR of compound **11**

Mass spectra of compound **11**


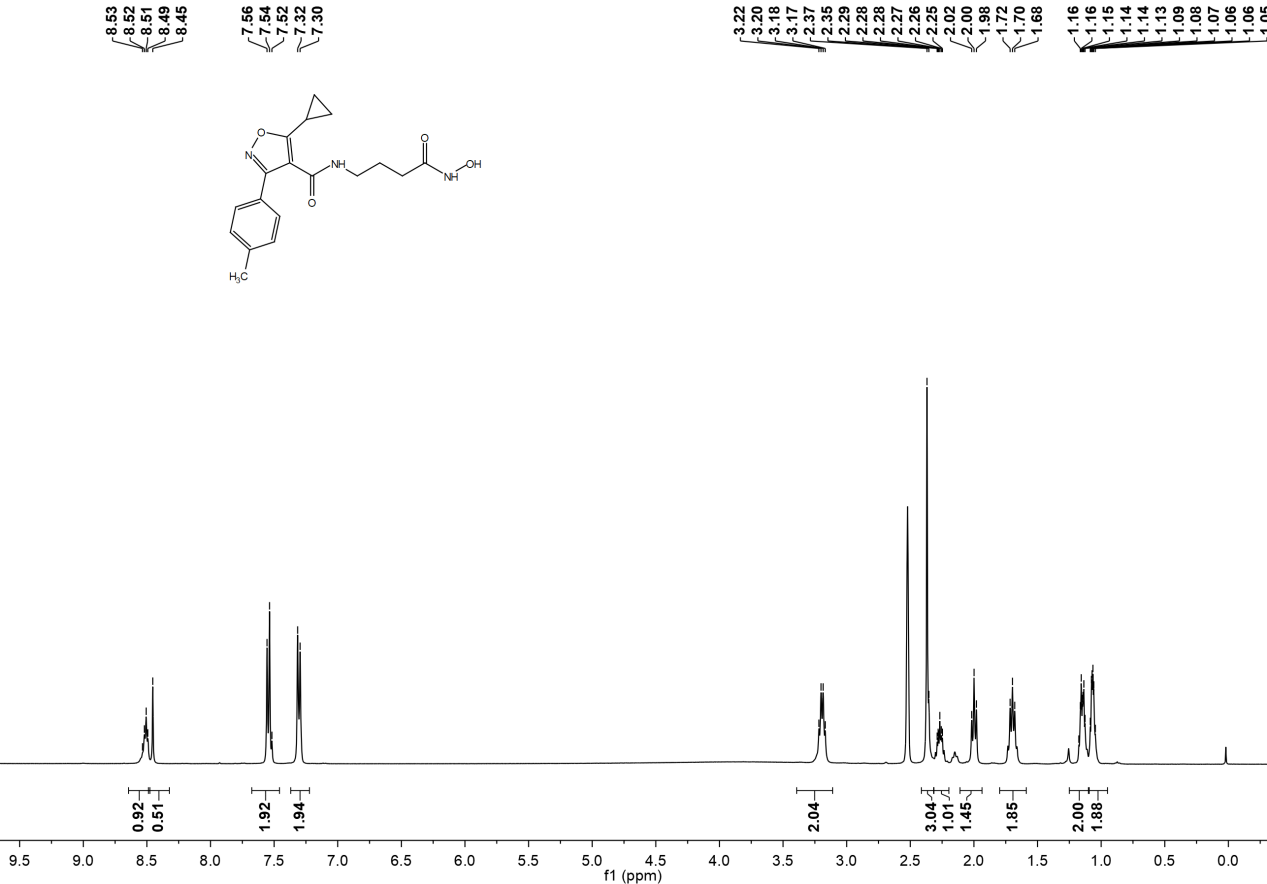


^1^H-NMR of compound **12**


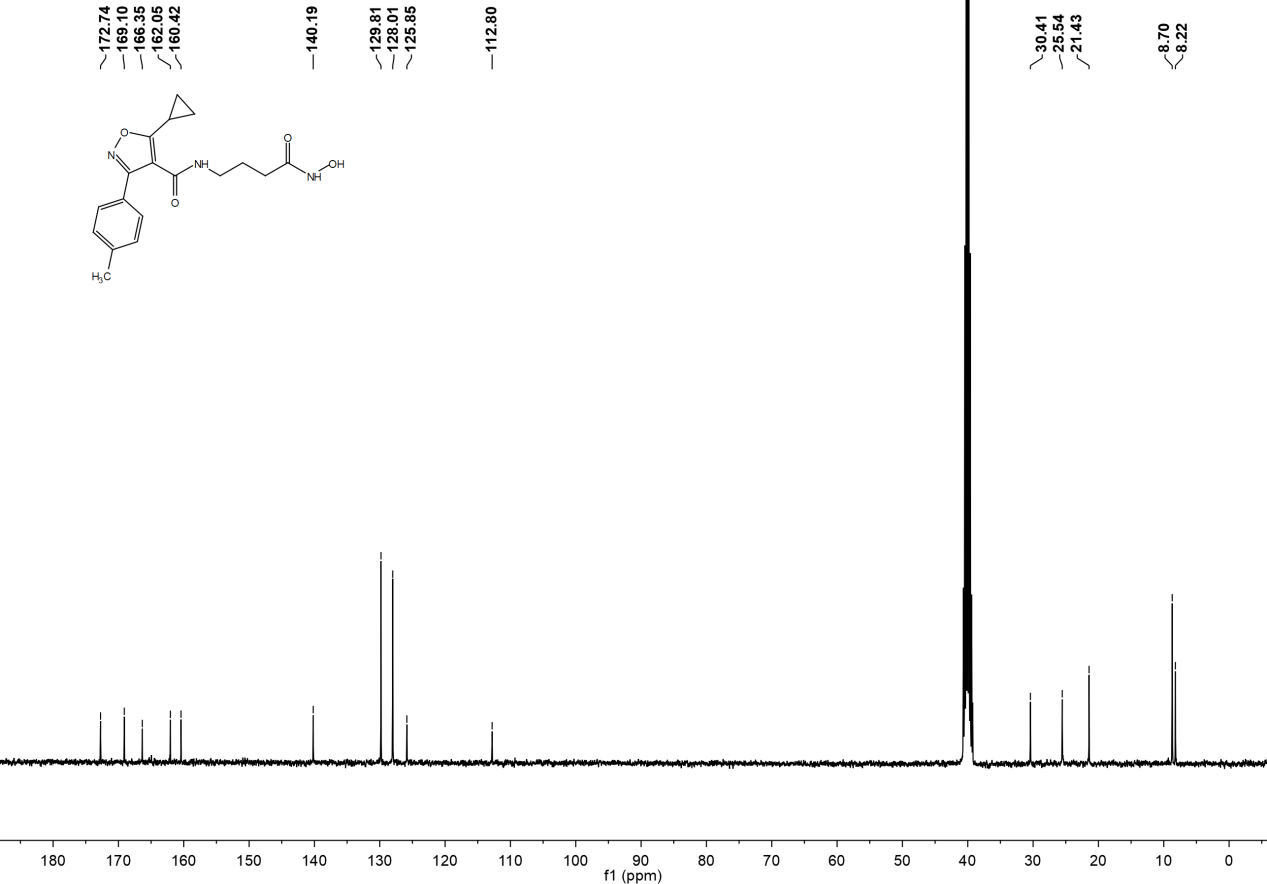


^13^C-NMR of compound **12**

Mass spectra of compound **12**


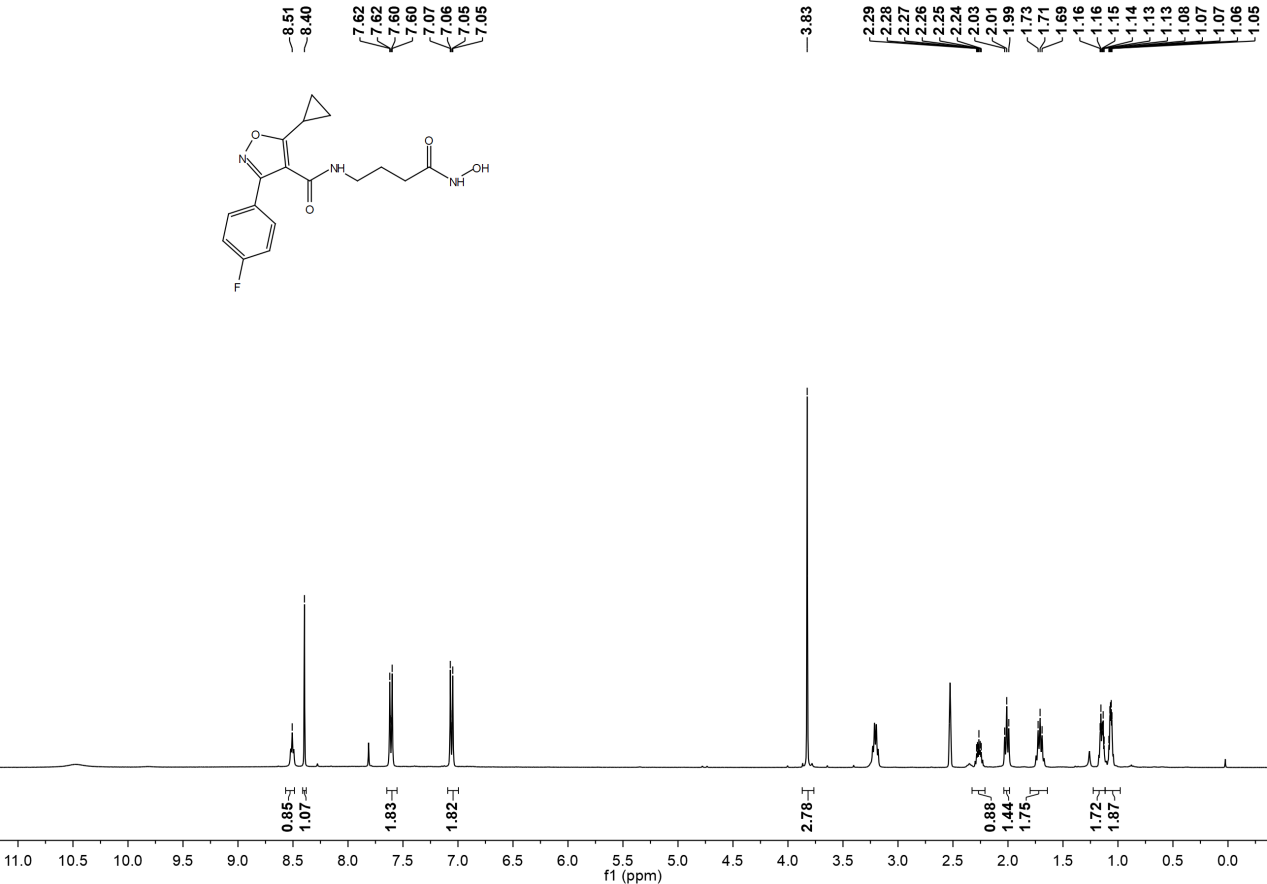


^1^H-NMR of compound **13**


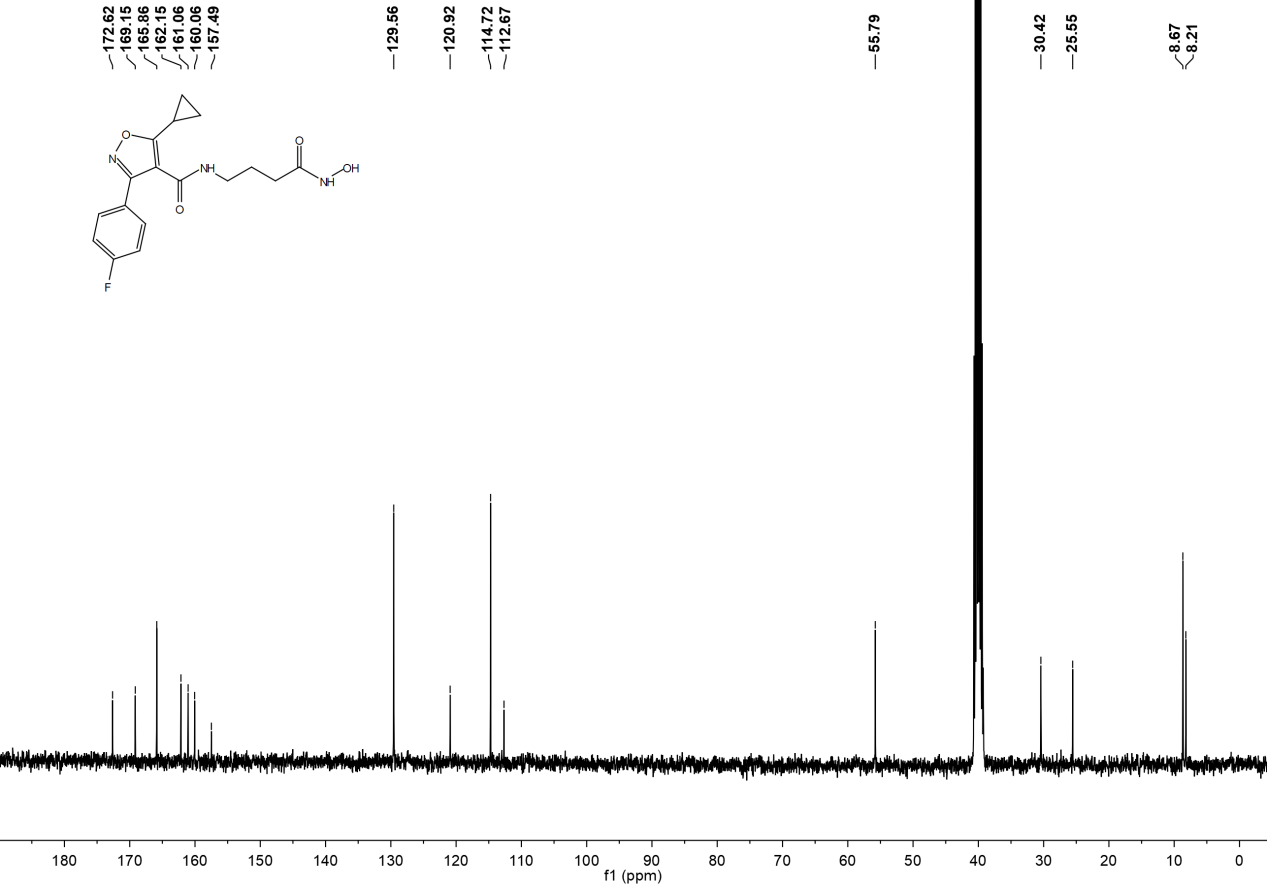


^13^C-NMR of compound **13**


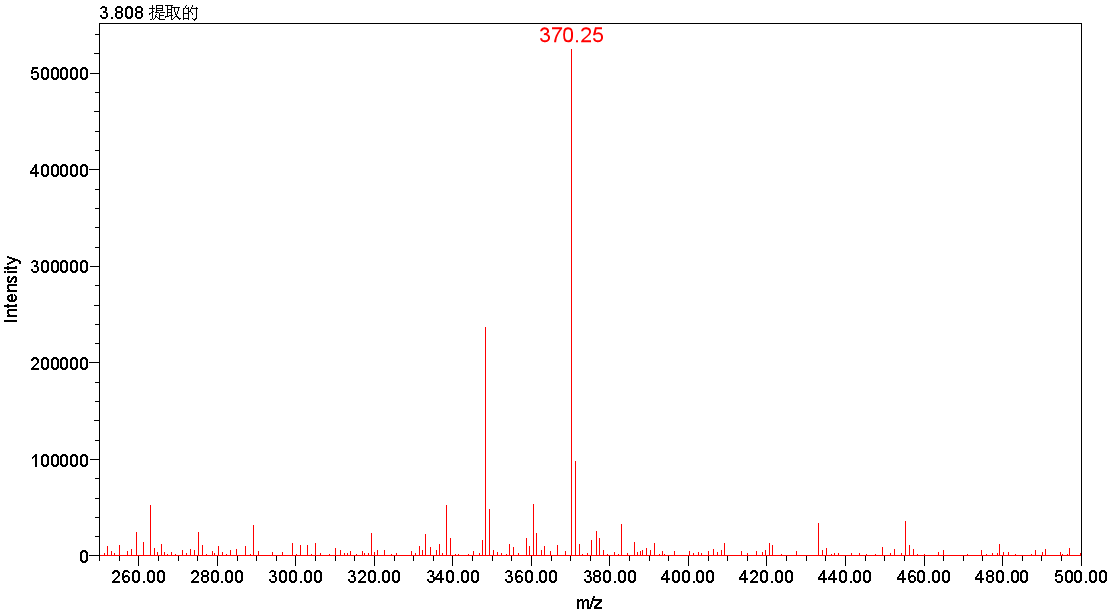


Mass spectra of compound **13**


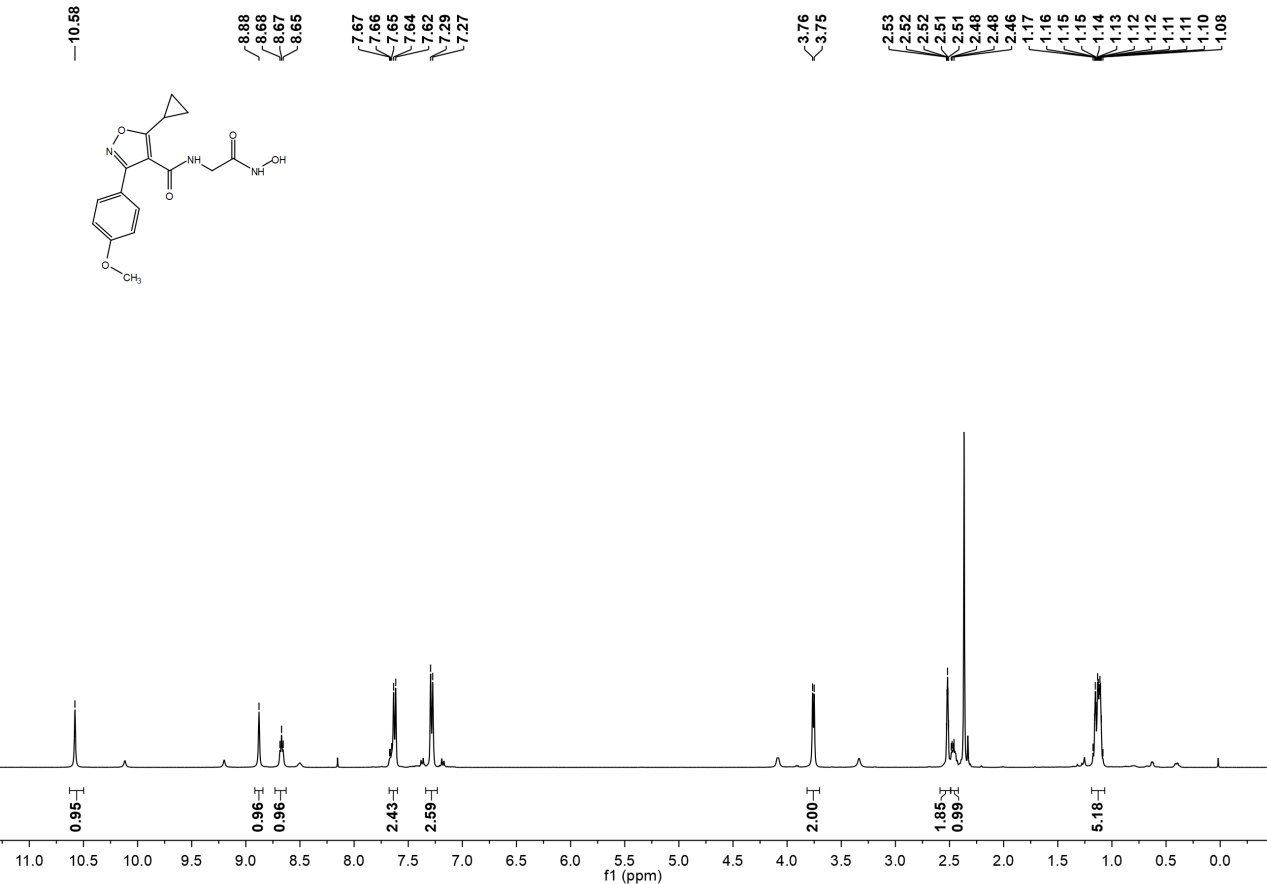


^1^H-NMR of compound **14**


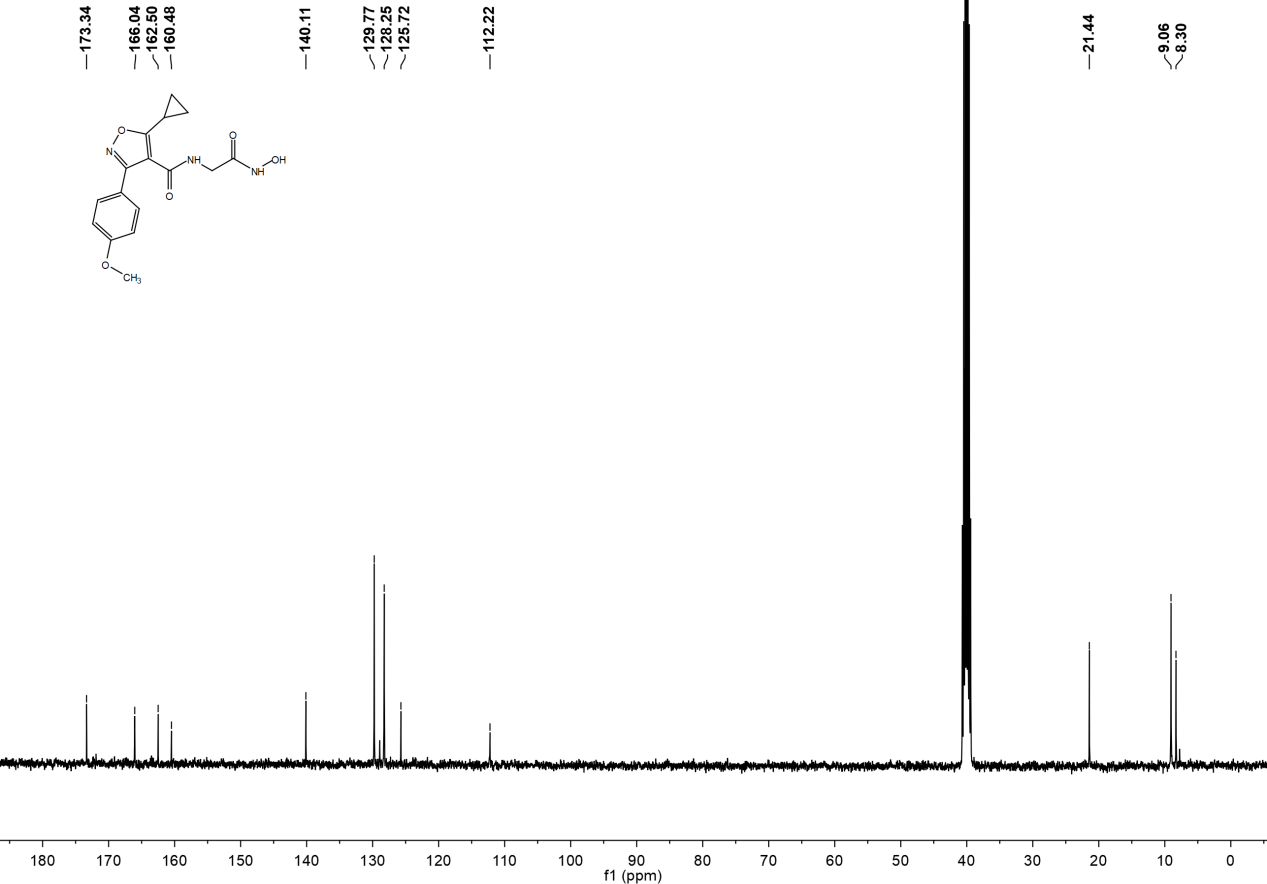


^13^C-NMR of compound **14**


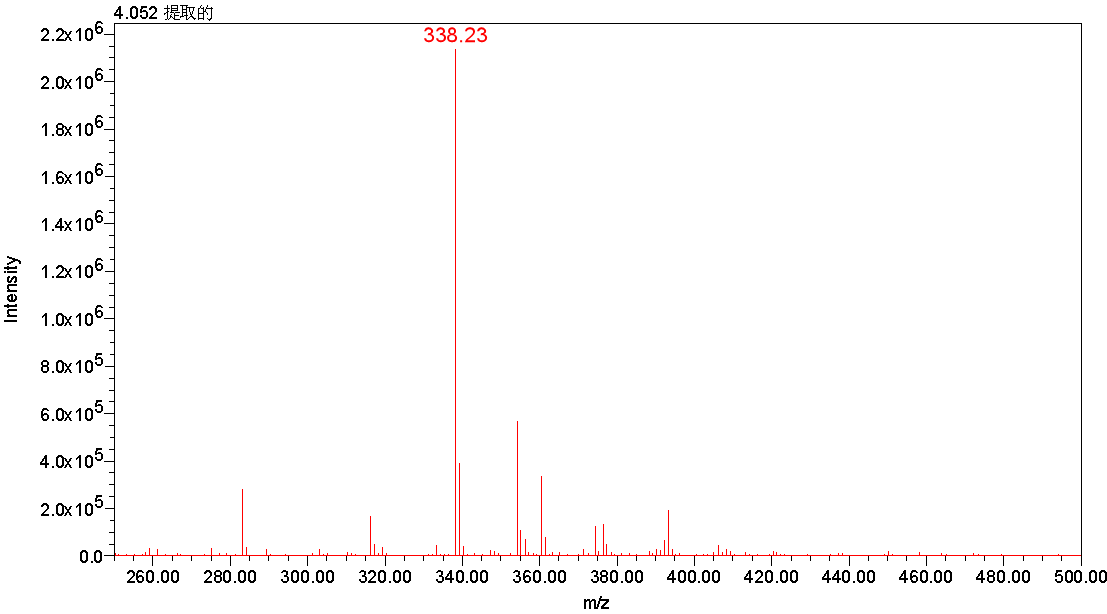


Mass spectra of compound **14**


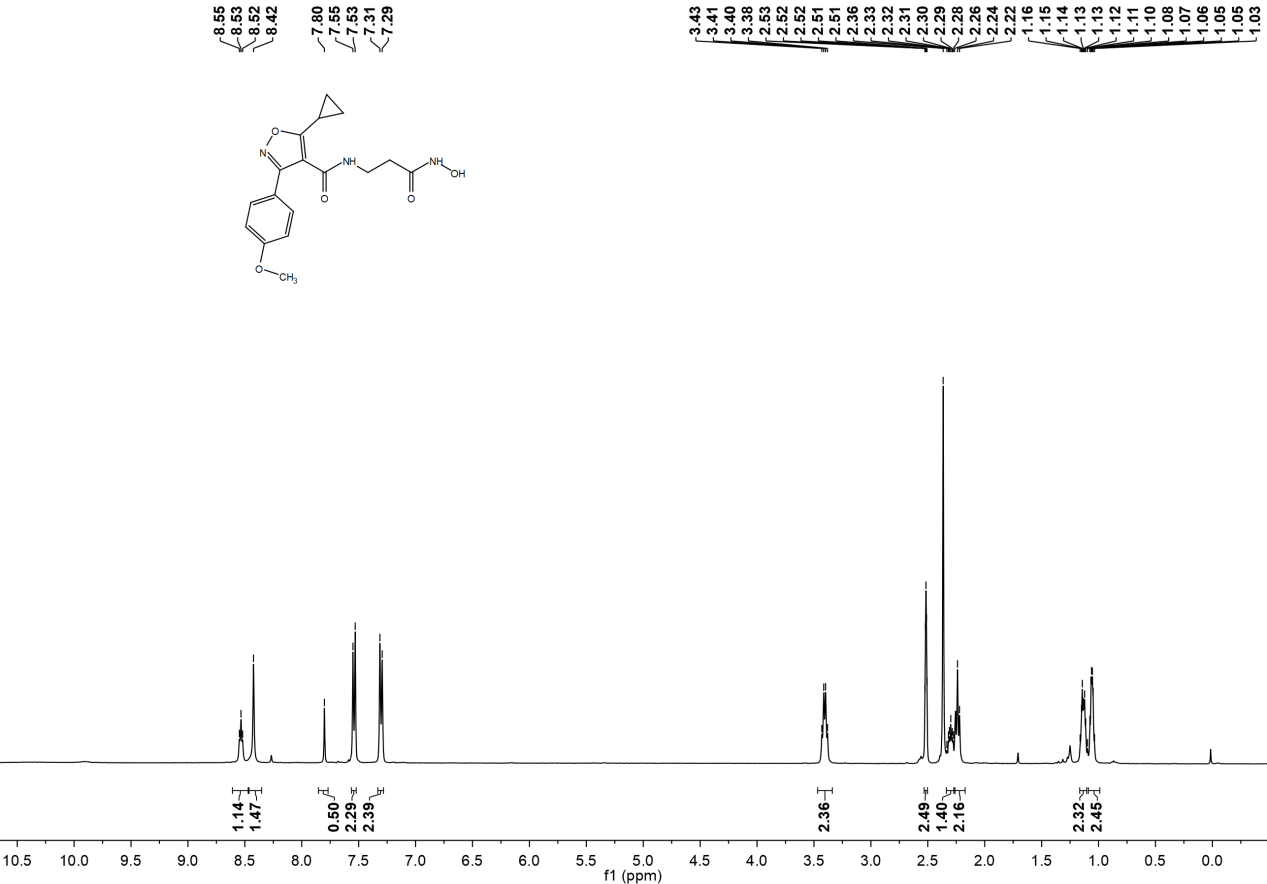


^1^H-NMR of compound **15**


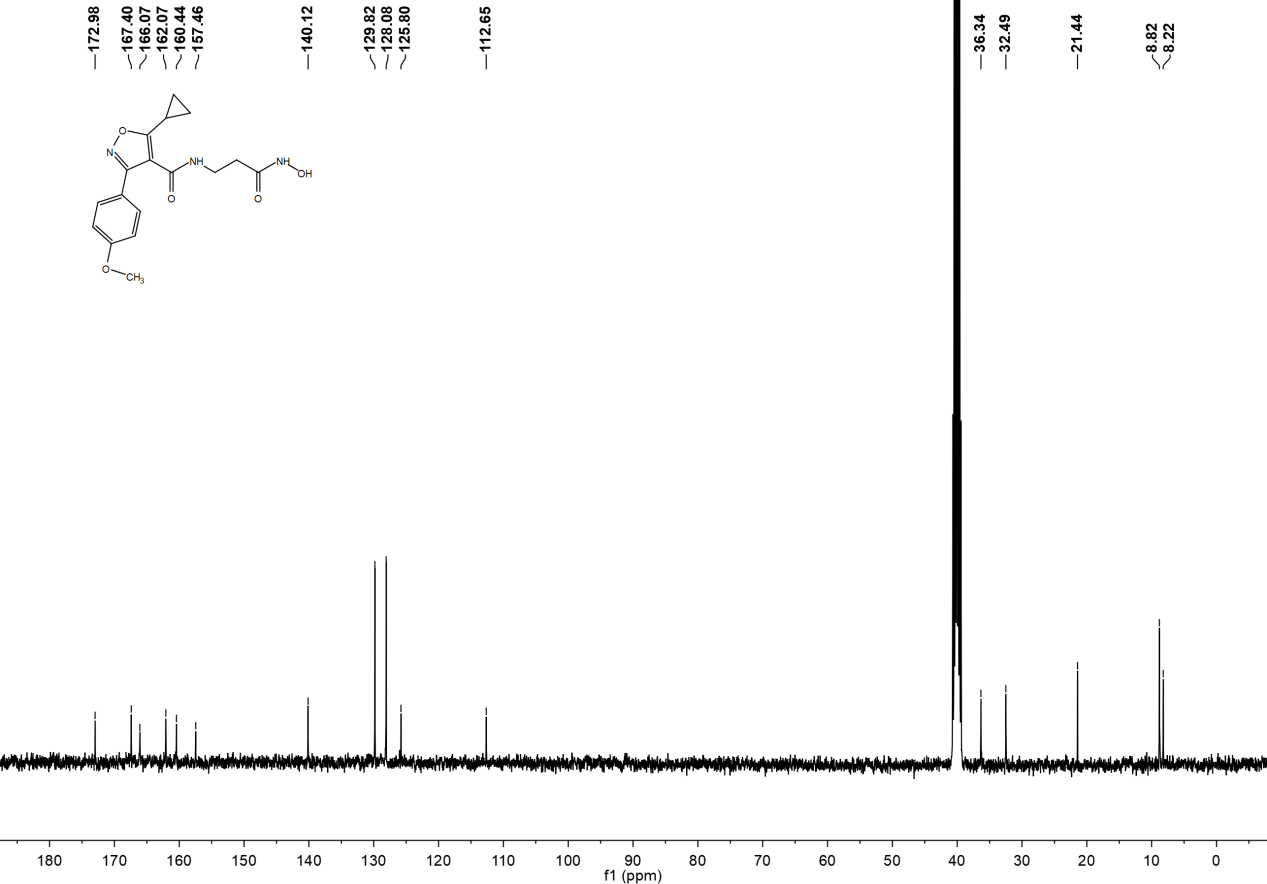


^13^C-NMR of compound **15**


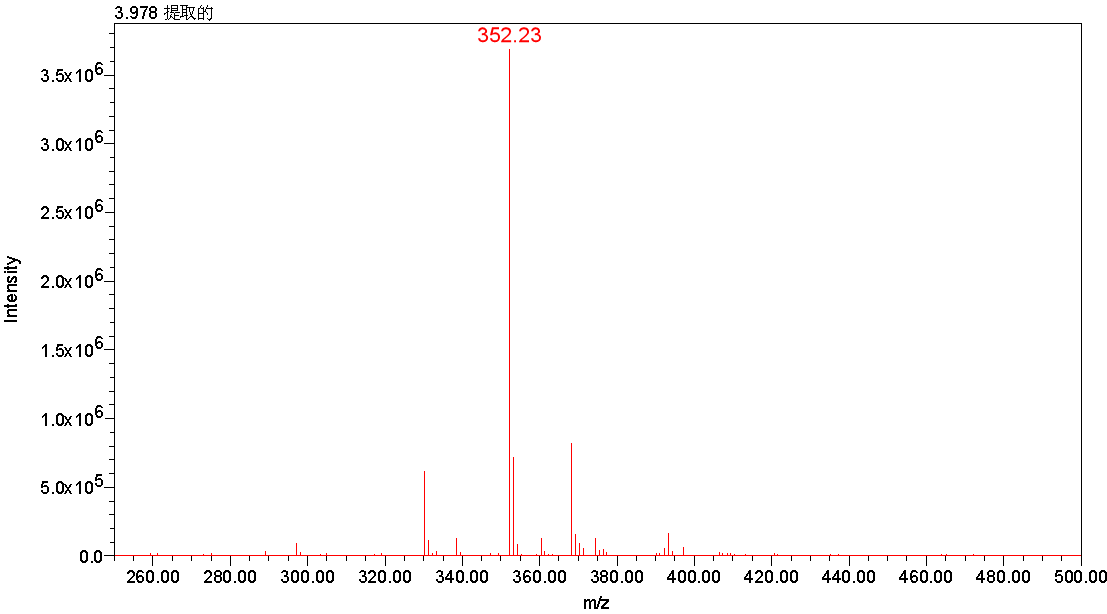


Mass spectra of compound **15**


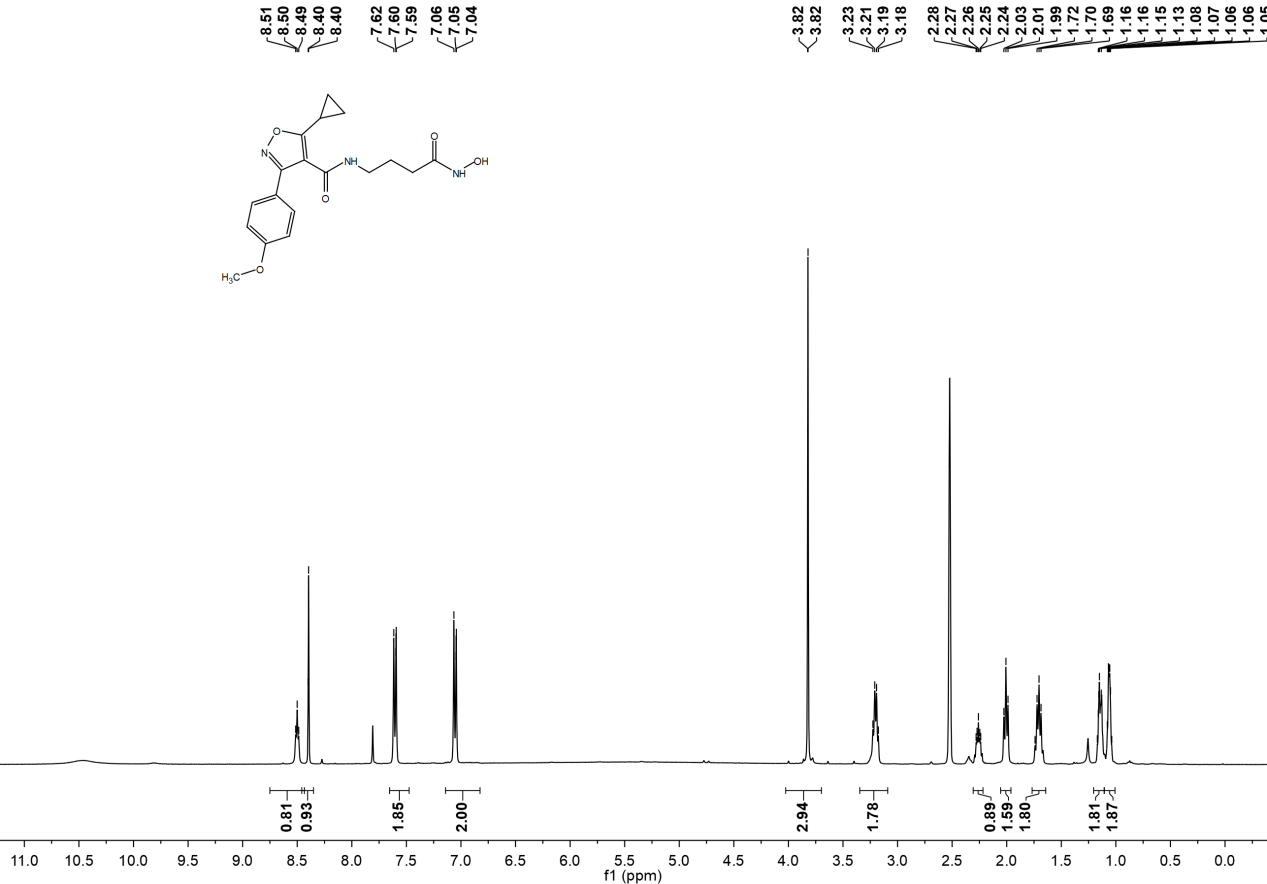


^1^H-NMR of compound **16**


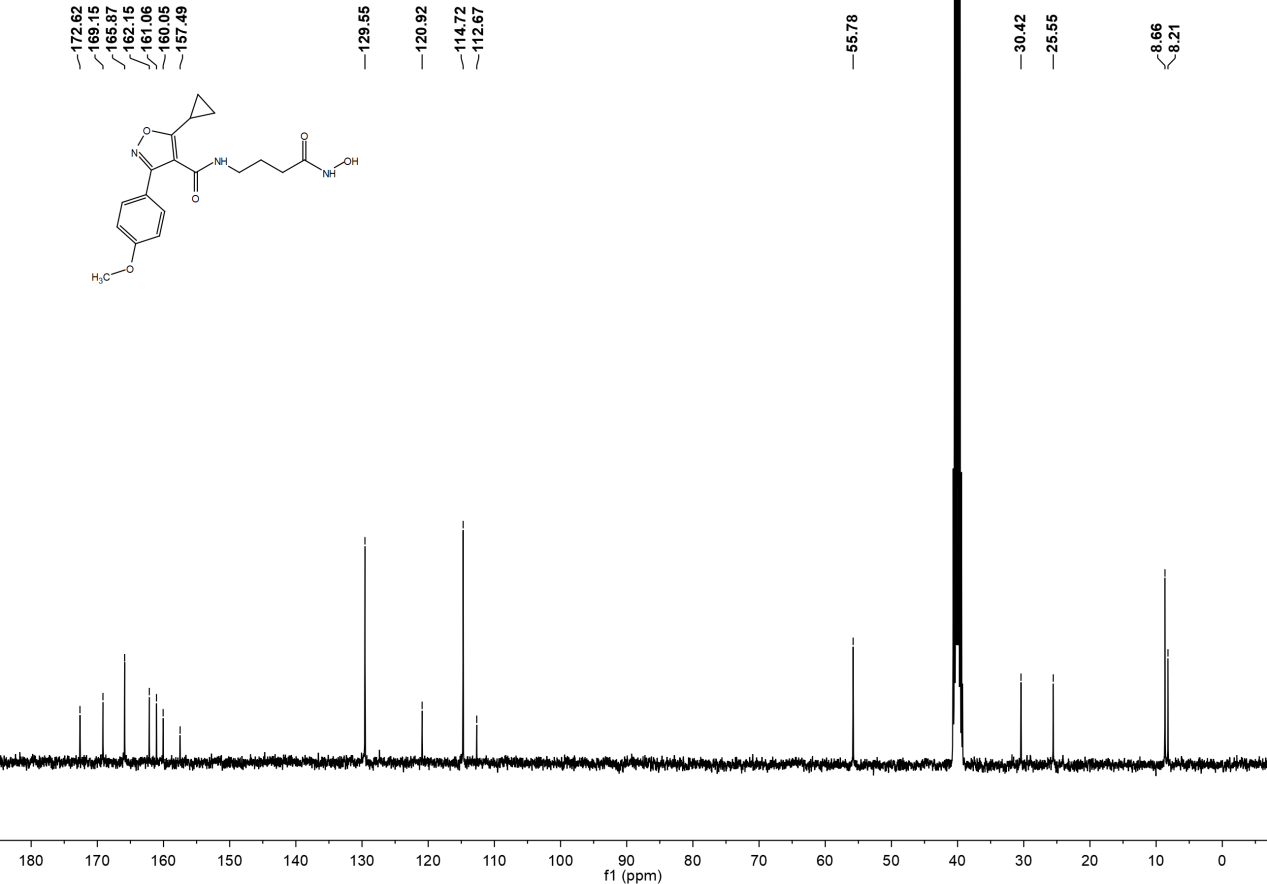


^13^C-NMR of compound **16**

Mass spectra of compound **16**


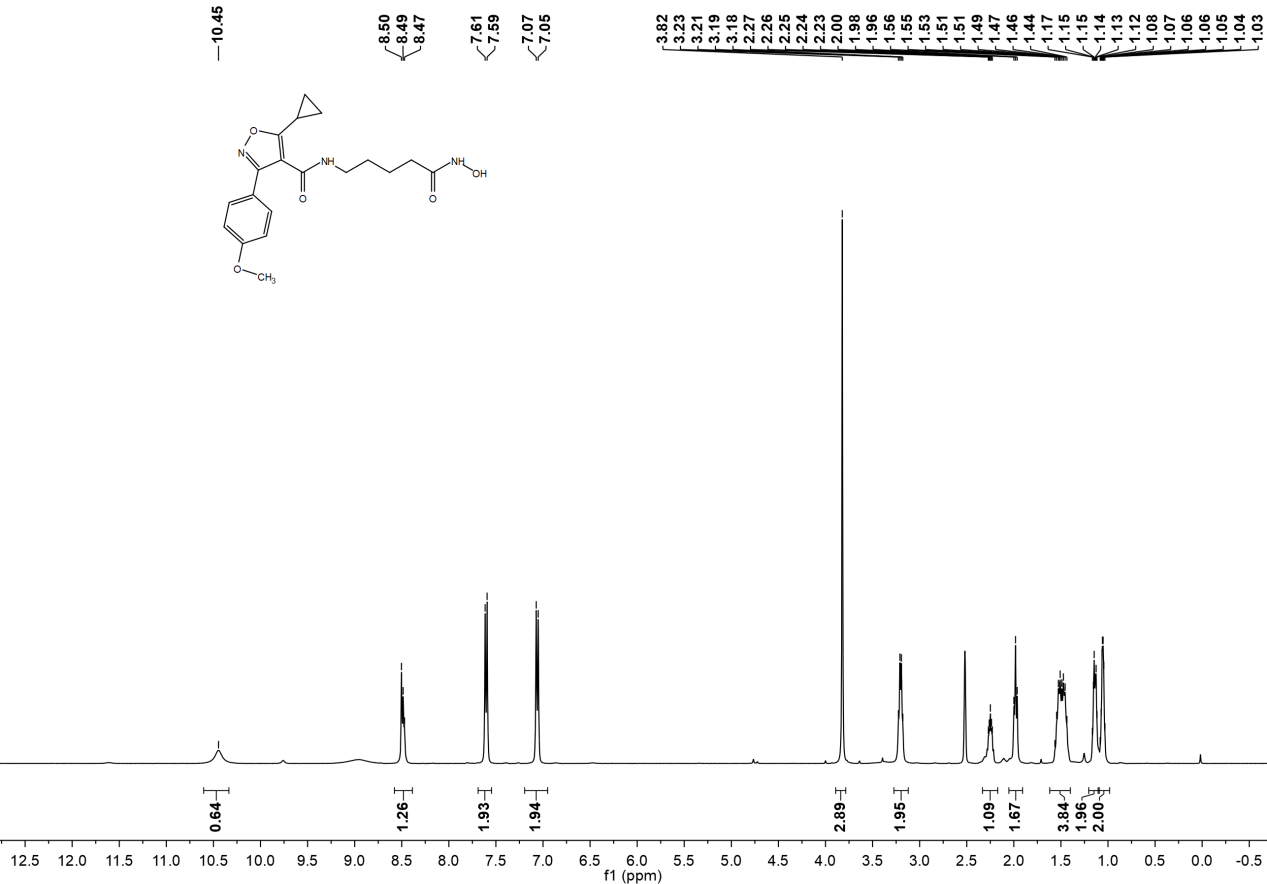


^1^H-NMR of compound **17**


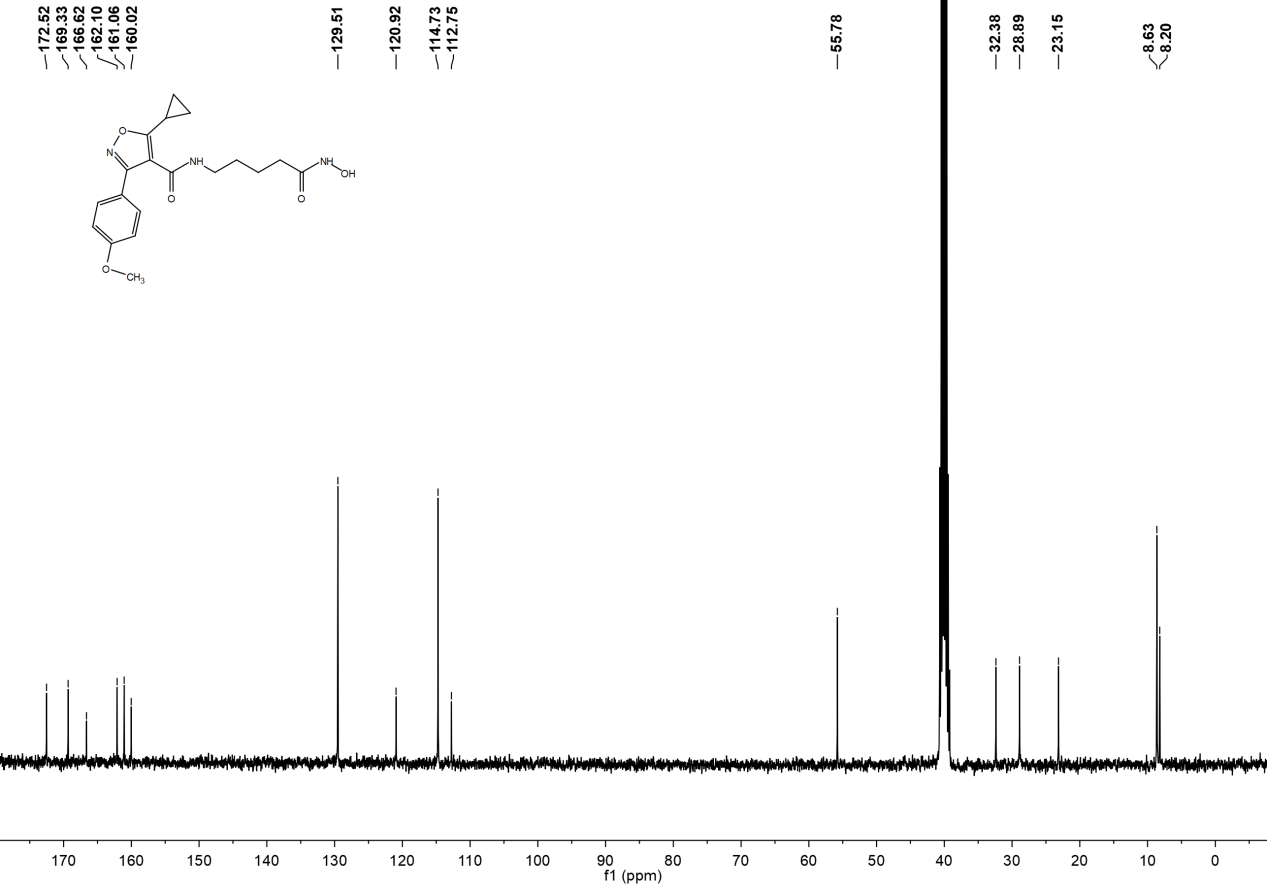


^13^C-NMR of compound **17**

Mass spectra of compound **17**


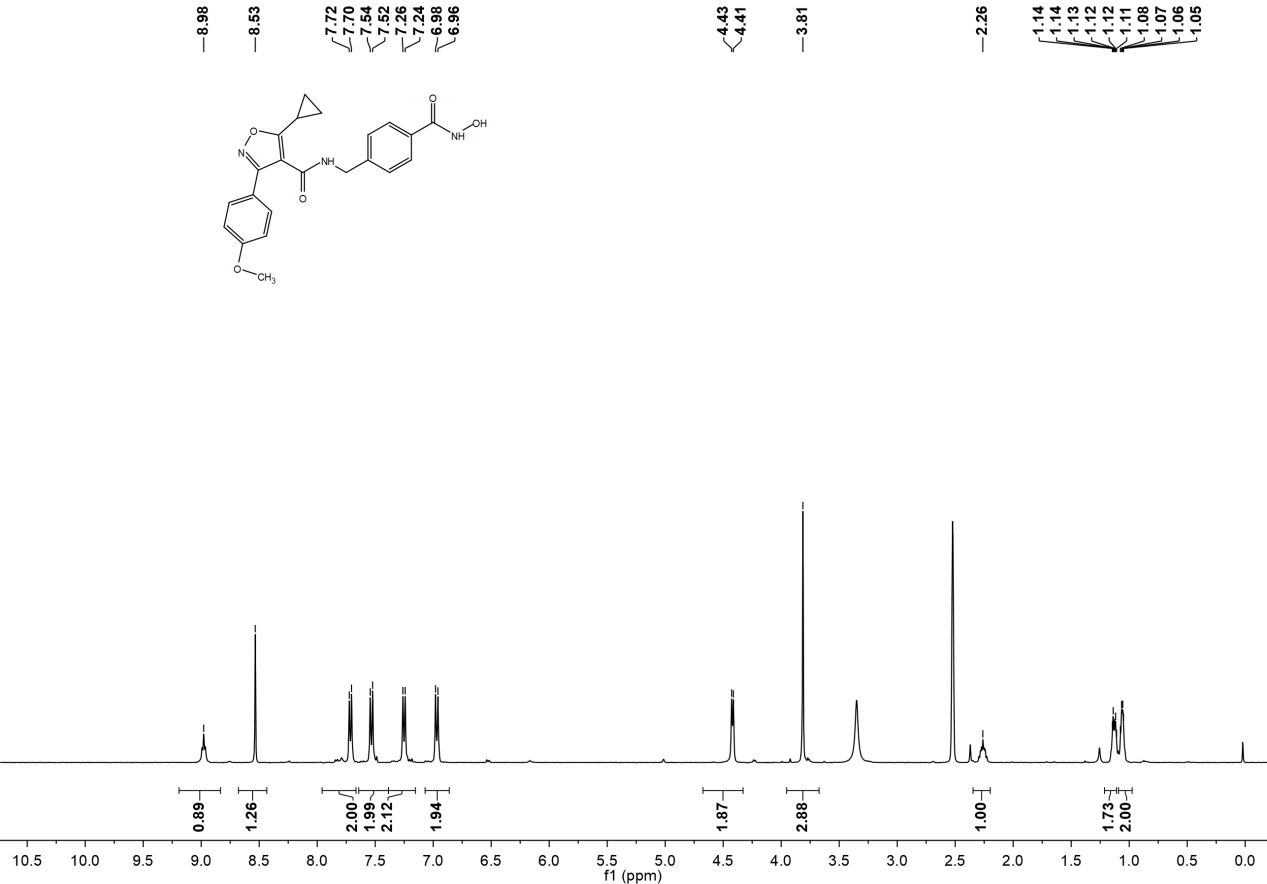


^1^H-NMR of compound **18**


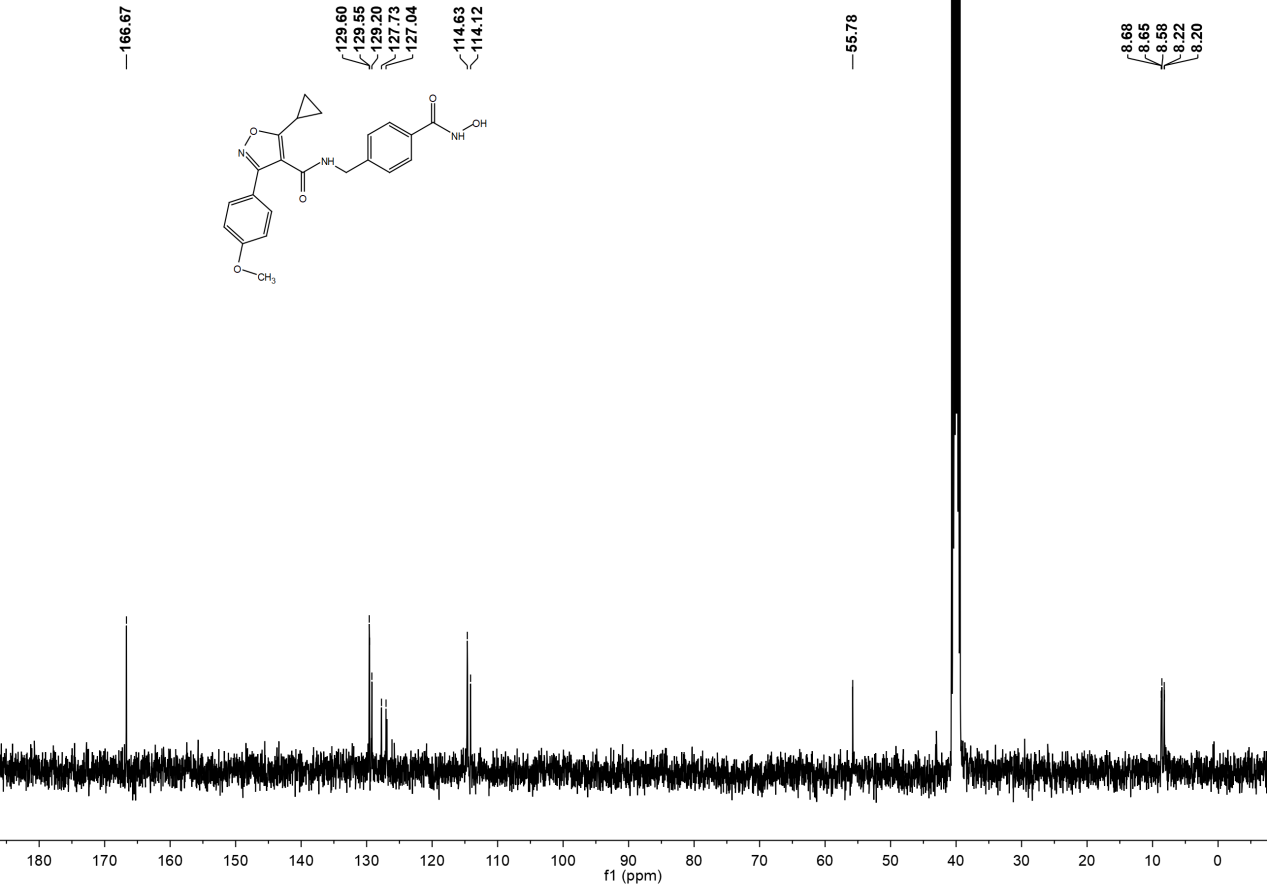


^13^C-NMR of compound **18**

Mass spectra of compound **18**

^1^H-NMR of compound **19**


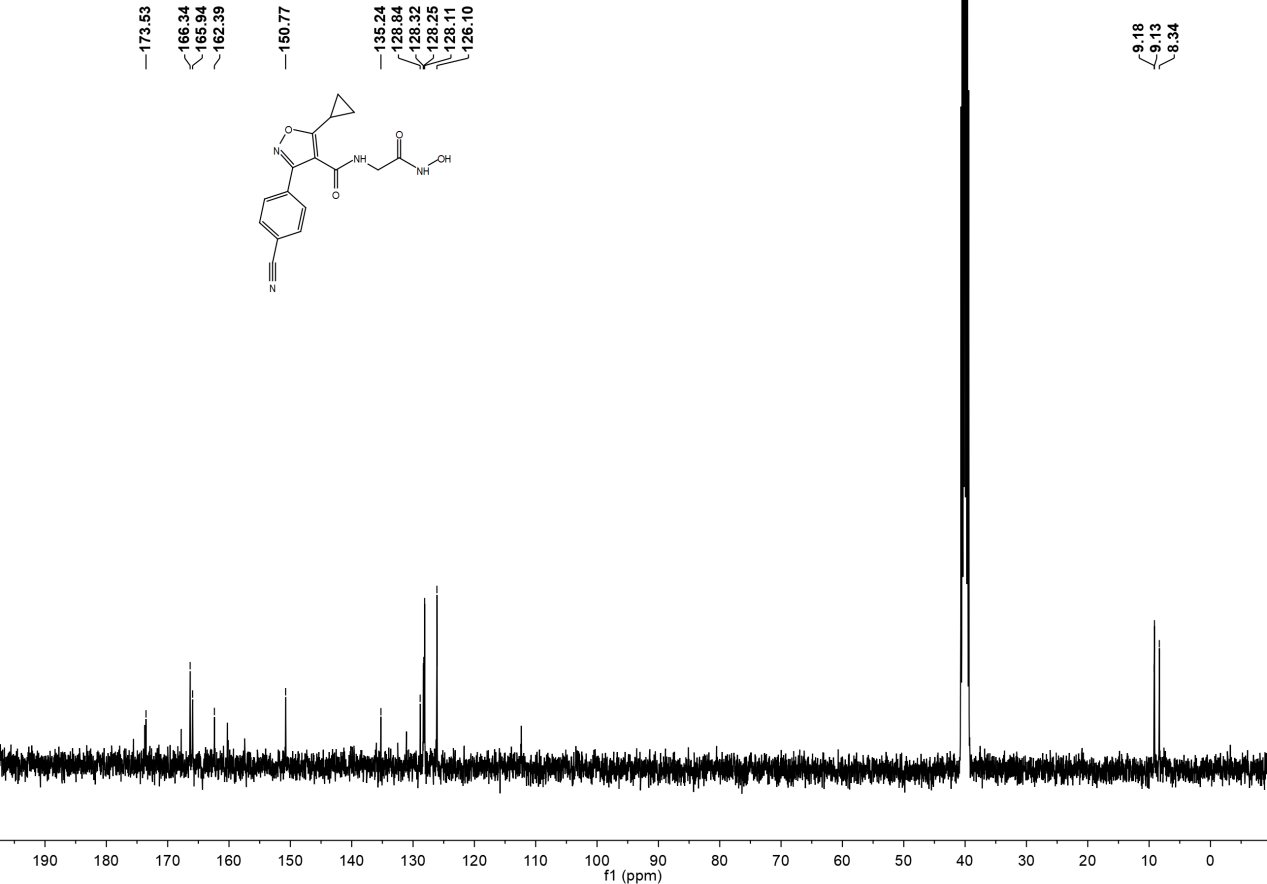


^13^C-NMR of compound **19**


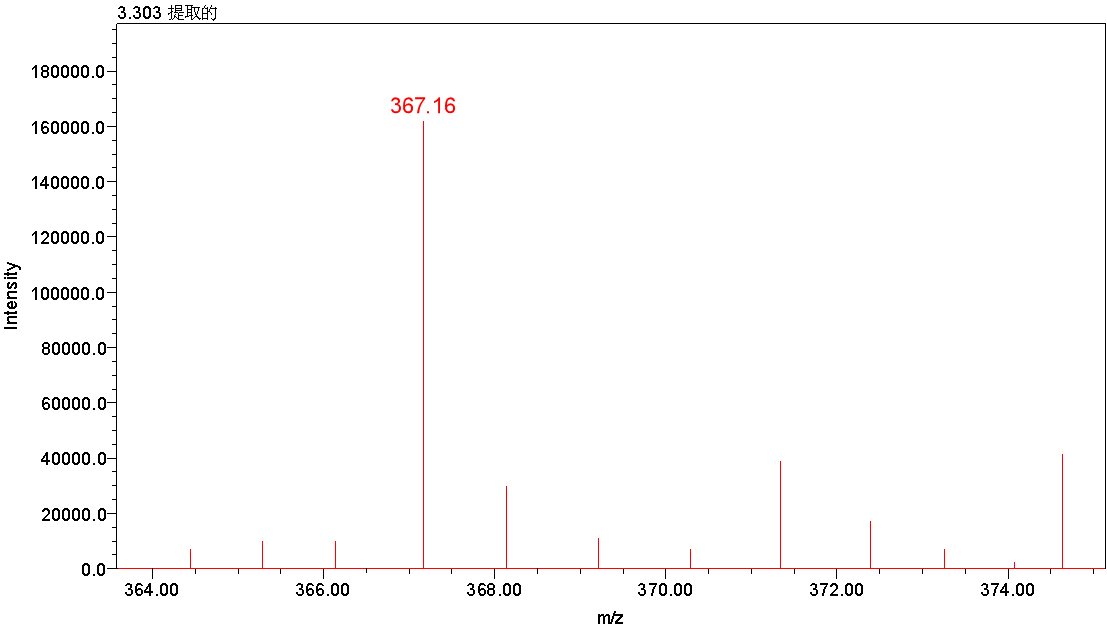


Mass spectra of compound **19**


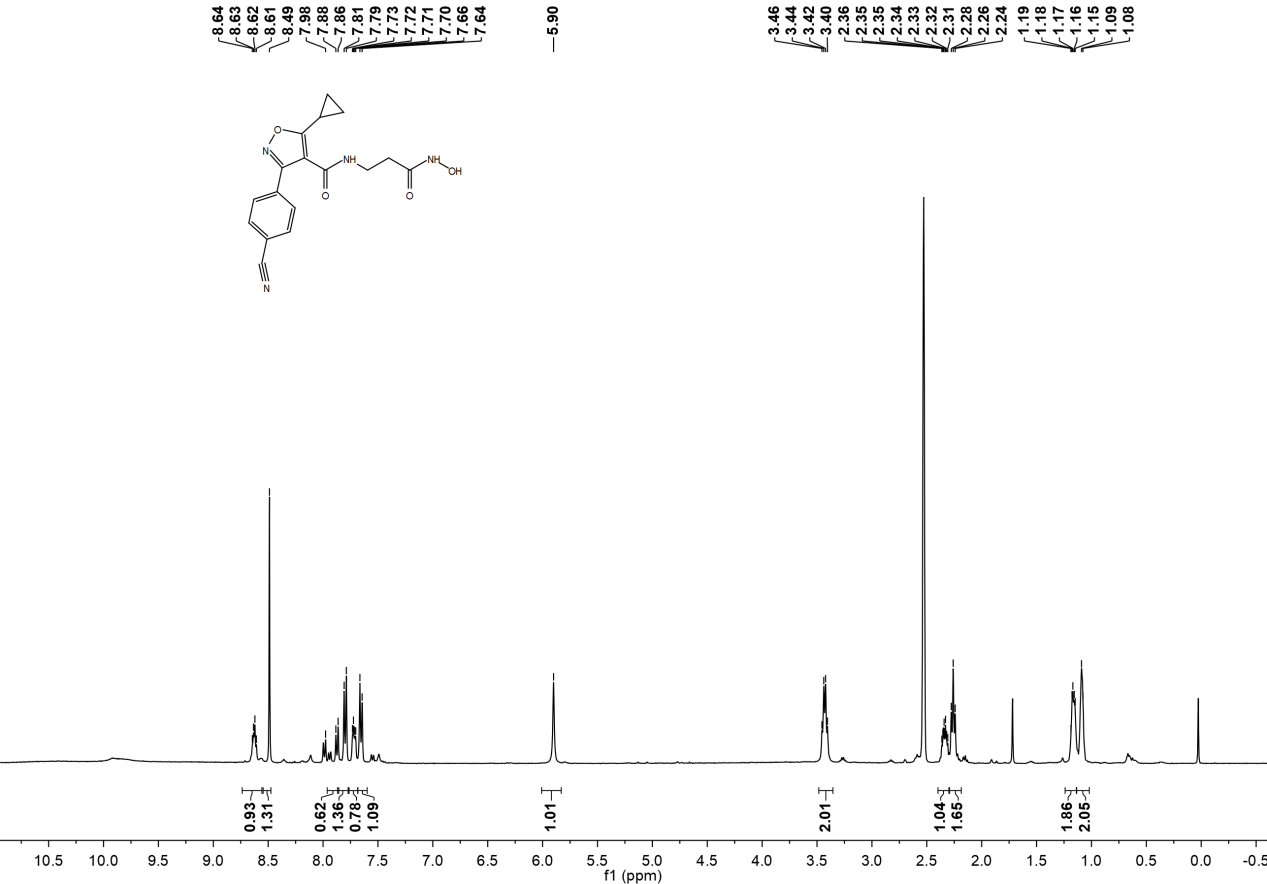


^1^H-NMR of compound **20**


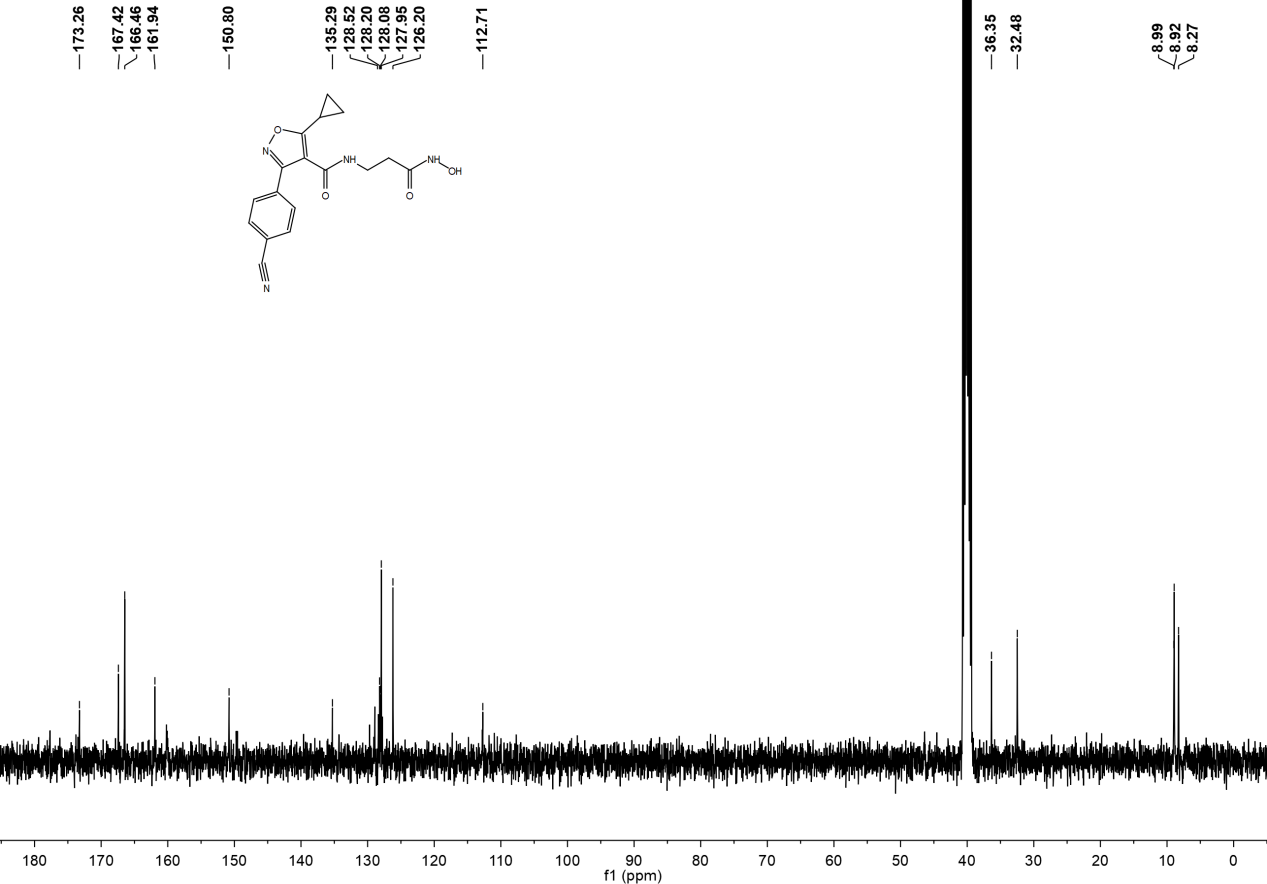


^13^C-NMR of compound **20**


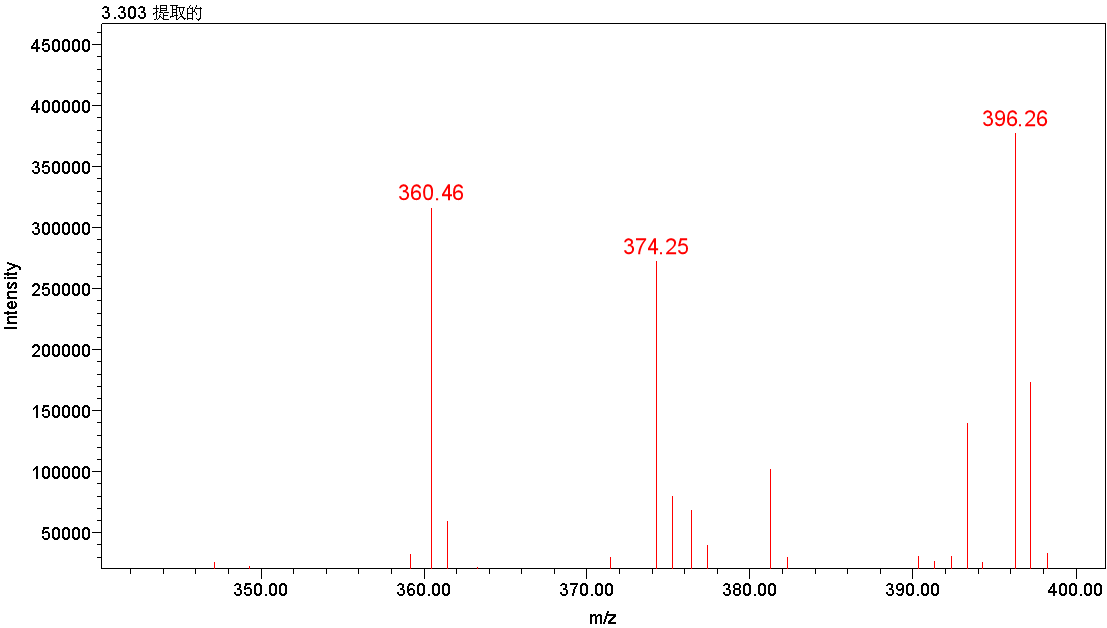


Mass spectra of compound **20**


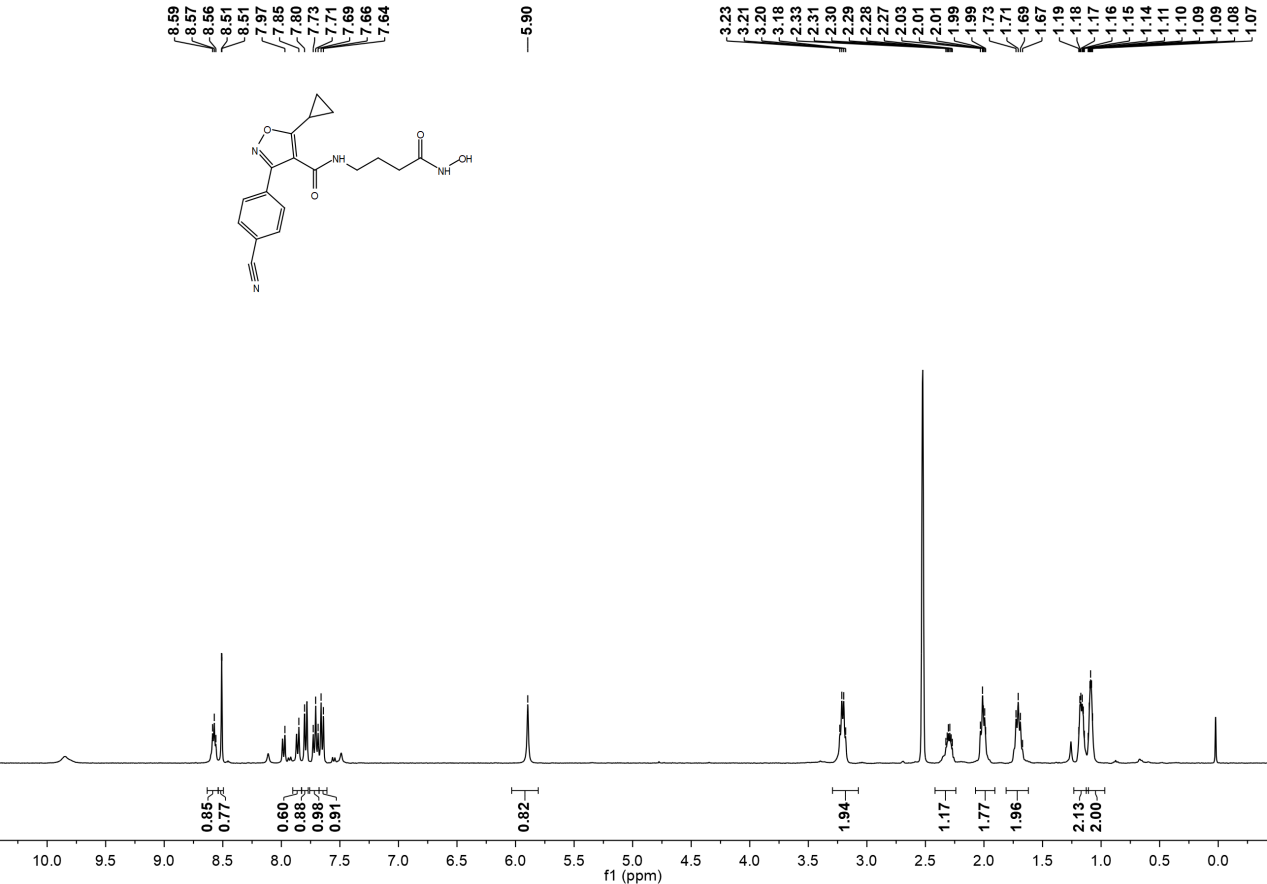


^1^H-NMR of compound **21**


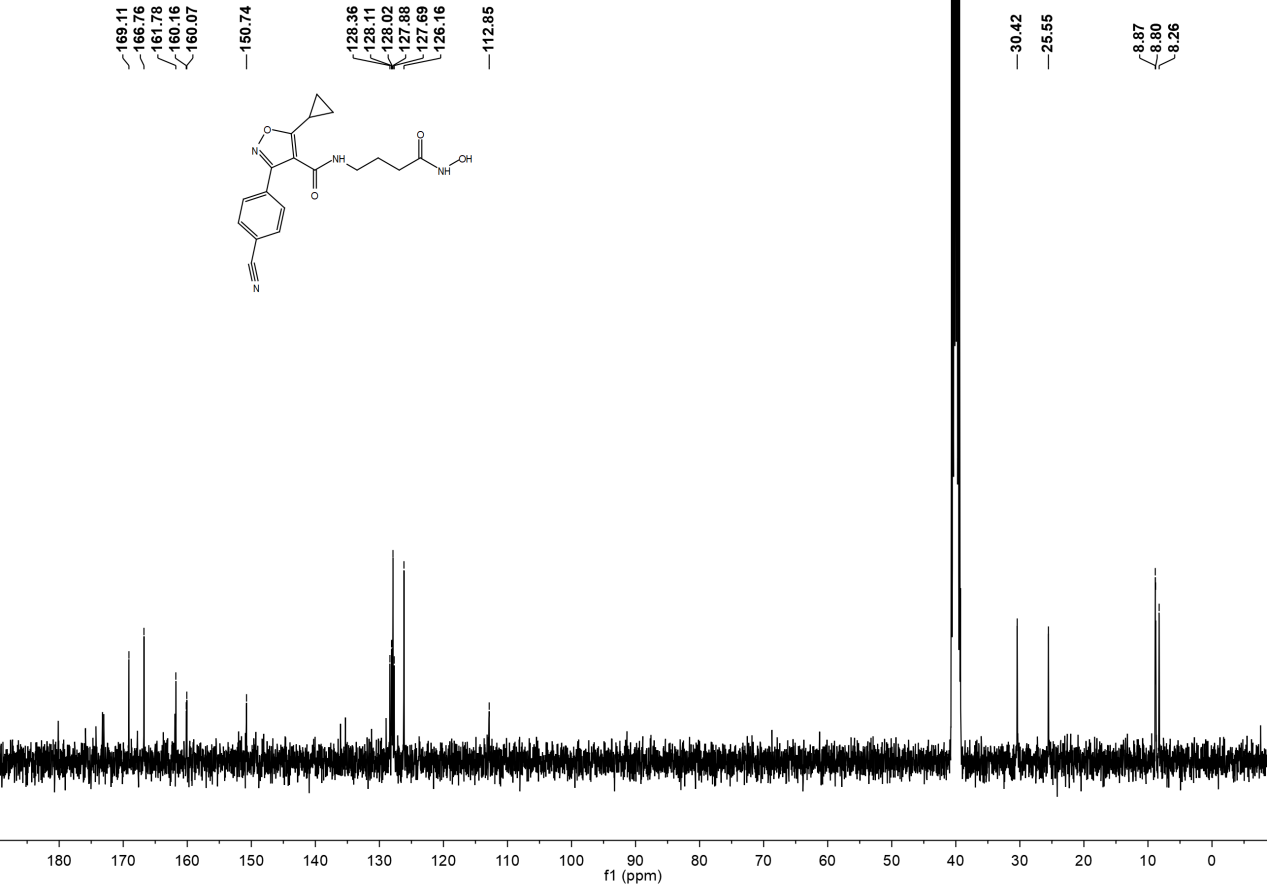


^13^C-NMR of compound **21**


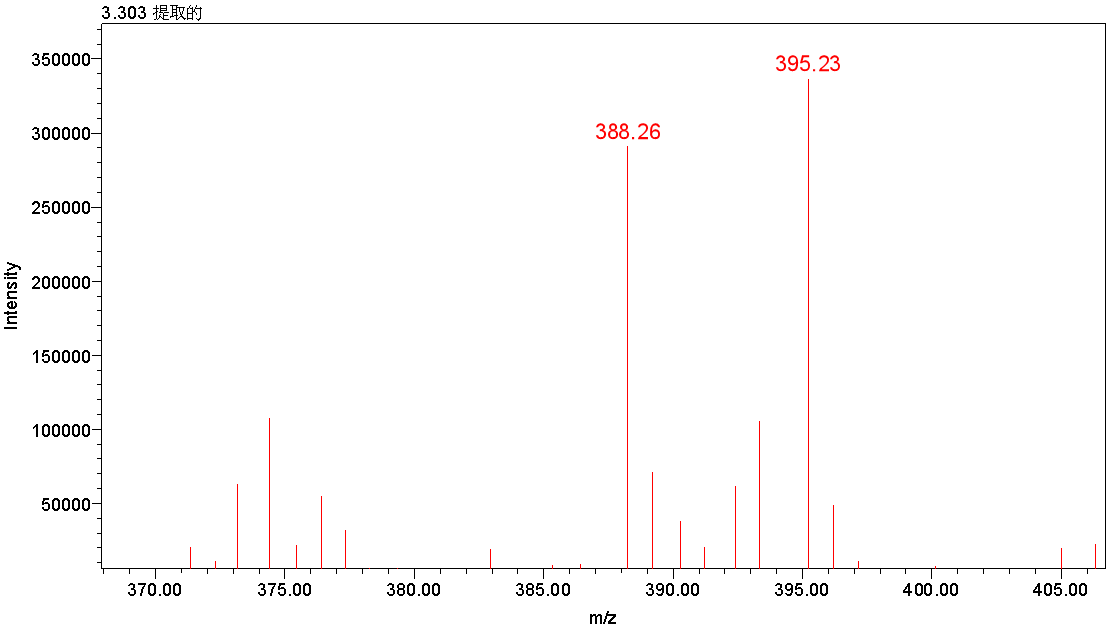


Mass spectra of compound **21**


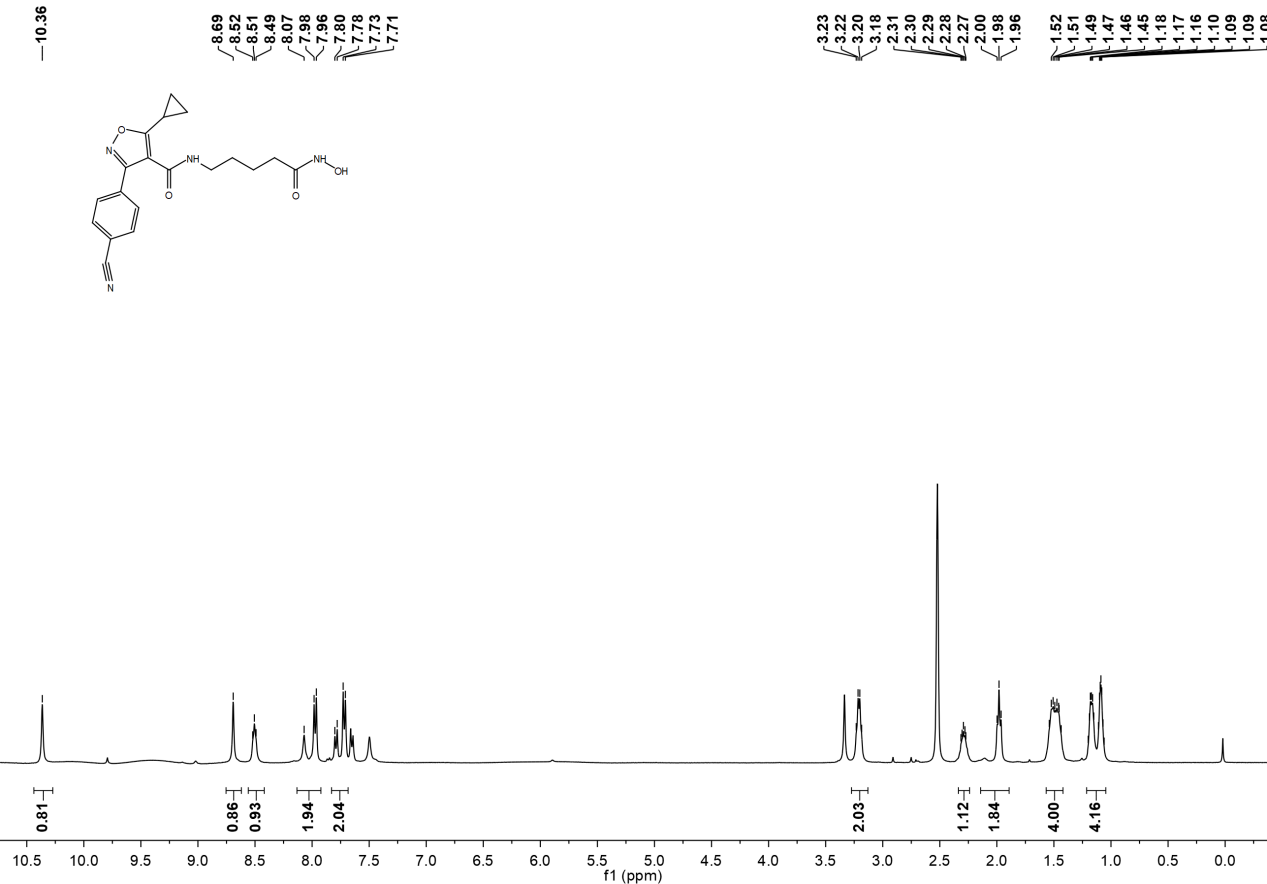


^1^H-NMR of compound **22**


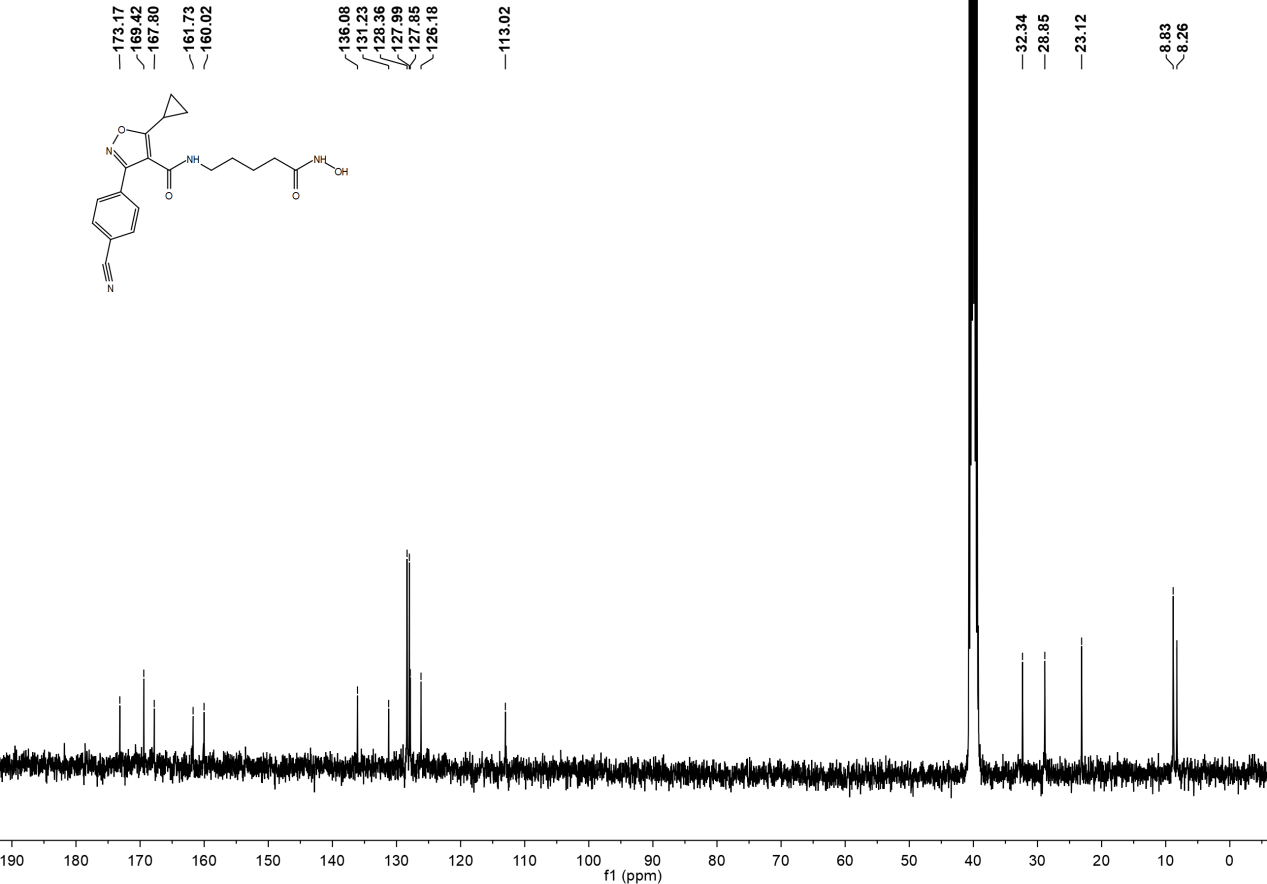


^13^C-NMR of compound **22**


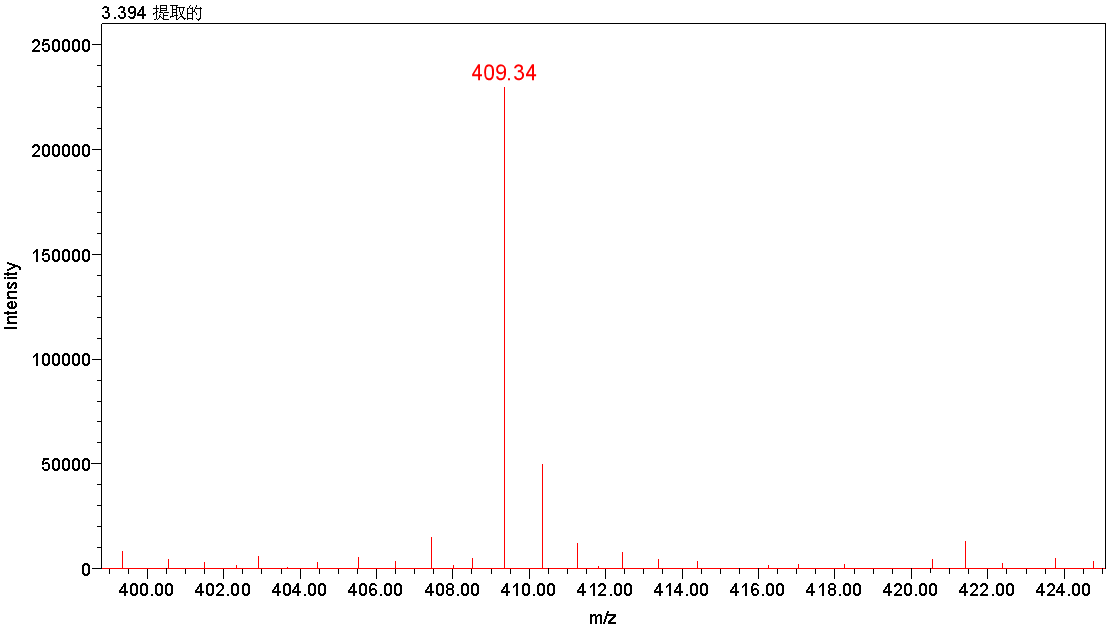


Mass spectra of compound **22**
