# Supplementary material for: Design, synthesis, and biological evaluation of phenylisoxazole-based histone deacetylase inhibitors
Source: PLoS One. 2025 Nov 5;20(11):e0334632. doi: 10.1371/journal.pone.0334632 (PMC12588503; doi:10.1371/journal.pone.0334632)
Supplement: Attachment — Submitted filename: PONE-D-25-33411.pdf [file pone.0334632.s002.pdf]

Manuscript Title: Design, synthesis, and biological evaluation of phenylisoxazole-based histone deacetylase inhibitors

Manuscript ID: PONE-D-25-33411

General Comments: The design, manufacturing, and pharmacological assessment of phenylisoxazole-based histone deacetylase (HDAC) inhibitors that specifically target HDAC1 are thoroughly examined in this publication. In silico modelling, synthetic chemistry, SAR analysis, drug-likeness assessment, and biological activity evaluation are all integrated in this well-structured work, which offers important insights into this unique scaffold.

Strengths and Significance:

1. Novel Scaffold Development: An important addition to the chemical diversity of HDAC inhibitors is the discovery and refinement of phenylisoxazole derivatives as HDAC1 inhibitors, which go beyond traditional hydroxamic acids and benzamides. Potential benefits in terms of toxicity profiles and selectivity are provided by the phenylisoxazole core.

2. Rational Design and SAR: The alterations at R1 and R2 positions were successfully directed by the structure-based drug design approach, which was aided by molecular docking. This resulted in compounds with increased activity, most notably compound 17. The comprehensive SAR analysis shows that linker length at R2 has a major impact on activity, with longer chains—like butyl—providing greater potency.

3. Synthetic Accessibility: Using widely accessible starting materials and conventional reactions, the synthetic approach outlined is simple and allows for additional optimisation and scalability.

4. Biological Evaluation: Potential therapeutic relevance is demonstrated by the combination of antiproliferative studies on prostate cancer PC3 cells and enzymatic HDAC1 inhibition assays. It is especially encouraging that WPMY-1 is selective for cancer cells rather than healthy prostate cells.

5. Drug-Likeness and ADMET Predictions: Compound 17 and its derivatives have good drug-like profiles, according to the computational assessment of pharmacokinetic characteristics and cardiotoxicity risk (hERG inhibition).

All things considered, this work presents a novel scaffold for HDAC1 inhibition that shows promise for treating prostate cancer. It is admirable how logical design, synthetic chemistry, and biological assessment have been combined. Future research addressing the aforementioned factors will enhance these compounds' translational potential and promote their advancement as medicinal agents.

Hence, I recommend this manuscript for publication.

Additionally, if possible the authors can add the below references. Thank you.

1. Cao, D., Zhou, X., Guo, Q., Xiang, M., Bao, M., He, B.,... Mao, X. (2024). Unveiling the role of histone deacetylases in neurological diseases: focus on epilepsy. Biomarker Research, 12(1), 142. doi: 10.1186/s40364-024-00687-6

2. Huang, T., Chen, Y., Zhao, Q., Wu, X., Li, H., Luo, X.,... Tang, N. (2025). Dual Regulation of Sprouty 4 Palmitoylation by ZDHHC7 and Palmitoyl-Protein Thioesterase 1: A Potential Therapeutic Strategy for Cisplatin-Resistant Osteosarcoma. Research, 8, 708. doi: 10.34133/research.0708
