# Supplementary material for: Design, synthesis, and biological evaluation of phenylisoxazole-based histone deacetylase inhibitors
Source: PLoS One. 2025 Nov 5;20(11):e0334632. doi: 10.1371/journal.pone.0334632 (PMC12588503; doi:10.1371/journal.pone.0334632)
Supplement: Attachment — Submitted filename: Reviewer comments of plos one.docx [file pone.0334632.s003.docx]

Manuscript Number: PONE-D-25-33411

Title: Design, synthesis, and biological evaluation of phenylisoxazole-based histone deacetylase inhibitors

The submitted manuscript describes the design, synthesis, and biological evaluation of phenylisoxazole-based histone deacetylase (HDAC) inhibitors as potential anticancer agents. While the topic aligns with the scope of the journal, the work presents limited novelty and requires substantial revisions before it can be reconsidered for publication. The authors are advised to address the following major concerns:

1. Please rewrite the keywords in alphabetical order to ensure consistency with journal formatting guidelines.
2. The manuscript contains multiple grammatical and typographical errors. A thorough language revision is necessary. Specifically:

a) Replace the word "novel" with "new" throughout the manuscript,

b) Ensure that all captions for schemes, tables, and figures are clearly indicated in bold within the main text.

c) Replace the abbreviation "eq" with "equiv." for consistency and clarity.

3. The HDAC1 inhibitory activity of Vorinostat (used as the positive control) should be included in Table 1 for comparison with the synthesized compounds.
4. There is inconsistency in the use of compound codes throughout the manuscript. Please review all compound identifiers carefully and present them in a uniform format, especially in schemes and Table 1.
5. The general procedure provided for the synthesis of compounds **6a–6p** uses 5% DCM: MeOH as the eluent. However, considering the structural variation among these compounds, it is unlikely that a single eluent system would be adequate for the purification of all derivatives. It is recommended to describe each reaction step individually, including the exact quantities of reagents and detailed purification methods for each compound.
6. Please verify and correct the M/Z values for compounds **19, 21, and 22**. Ensure that all reported mass data are accurate and correspond to the correct molecular formulas
